# How Pain-Related Facial Expressions Are Evaluated in Relation to Gender, Race, and Emotion

**DOI:** 10.1007/s42761-023-00181-6

**Published:** 2023-03-03

**Authors:** Troy C. Dildine, Carolyn M. Amir, Julie Parsons, Lauren Y. Atlas

**Affiliations:** 1 National Center for Complementary and Integrative Health, National Institutes of Health, 10, Center Drive, Bethesda, MD 20892 USA; 2 Department of Clinical Neuroscience, Karolinska Institute, 171 77 Solna, Sweden; 3 National Institute of Mental Health, National Institutes of Health, Bethesda, MD 20892 USA; 4 National Institute on Drug Abuse, National Institutes of Health, Baltimore, MD 21224 USA

**Keywords:** Health inequities, Facial expressions, Emotion recognition, Pain assessment

## Abstract

Inequities in pain assessment are well-documented; however, the psychological mechanisms underlying such biases are poorly understood. We investigated potential perceptual biases in the judgments of faces displaying pain-related movements. Across five online studies, 956 adult participants viewed images of computer-generated faces (“targets”) that varied in features related to race (Black and White) and gender (women and men). Target identity was manipulated across participants, and each target had equivalent facial movements that displayed varying intensities of movement in facial action-units related to pain (Studies 1–4) or pain and emotion (Study 5). On each trial, participants provided categorical judgments as to whether a target was in pain (Studies 1–4) or which expression the target displayed (Study 5) and then rated the perceived intensity of the expression. Meta-analyses of Studies 1–4 revealed that movement intensity was positively associated with both categorizing a trial as painful and perceived pain intensity. Target race and gender did not consistently affect pain-related judgments, contrary to well-documented clinical inequities. In Study 5, in which pain was equally likely relative to other emotions, pain was the least frequently selected emotion (5%). Our results suggest that perceivers can utilize facial movements to evaluate pain in other individuals, but perceiving pain may depend on contextual factors. Furthermore, assessments of computer-generated, pain-related facial movements online do not replicate sociocultural biases observed in the clinic. These findings provide a foundation for future studies comparing CGI and real images of pain and emphasize the need for further work on the relationship between pain and emotion.

**Supplementary Information:**

The online version contains supplementary material available at 10.1007/s42761-023-00181-6.

In the USA, medical providers underassess pain in socially marginalized patients (e.g., women and racial and ethnic minorities; Anderson et al., 2009; Green et al., 2003; LeResche, 2011; Todd et al., 1993). Clinicians and researchers alike typically assess pain using unidimensional self-report measures (e.g., the 0–10 Numerical Rating Scale), which should lead to standardized treatment (Vila et al., 2005). Yet, despite Black and women patients experiencing *more* pain in the lab and clinic, respectively (Kim et al., 2017; Mogil, 2012), they are conversely less likely to receive treatment and receive less potent treatments for pain (Cintron & Morrison, 2006; Mossey, 2011). This indicates that standardized pain measures do not lead to standardized pain treatments.

Researchers have begun to identify potential psychological mechanisms underlying non-standardized pain treatment and assessment (Drwecki et al., 2011; Hoffman et al., 2016). In particular, recent work indicates that sociocultural biases can influence the assessment of nonverbal pain behavior, i.e., pain-related facial movements (Hirsh et al., 2008; Mende-Siedlecki et al., 2019; Zhang et al., 2021) and that multiple sociodemographic factors (e.g., socioeconomic status and race; Summers et al., 2021) can impact the same pain assessment judgment. Although guidelines for nonverbal pain assessment exist for nonverbal patient populations (Payen et al., 2001), there is a lack of standard guidelines to assess nonverbal pain behaviors in verbal patient populations. This is particularly troubling as providers report looking at and integrating patient facial expressions during assessments (Johnson, 1977) and they believe facial expressions are more informative of their patients’ internal state compared to verbal self-reports (Poole & Craig, 1992). Thus, pain-related facial expression assessment is fundamental to pain evaluation and might provide insights on inequities in pain assessment.

Interestingly, although clinical pain assessments are biased against Black and women patients, findings from experimental assessments of facial expressions of pain have been mixed. Because most databases of pain-related facial expressions in actual patients are homogeneous (Dildine & Atlas, 2019), researchers use images of actors or computer-generated faces to manipulate identity (i.e., race and gender) and test whether sociodemographic factors impact pain assessment. While some studies observe biases that are consistent with disparities in pain assessment seen in clinical samples (Mende-Siedlecki et al., 2019; Zhang et al., 2021), others observe more pain attributed to faces depicting Black (Hirsh et al., 2008, 2009) or women patients ( Hirsh et al., 2008, 2009; Martel et al., 2011; Robinson & Wise, 2003) compared to White and men patients, respectively.

One important question is whether the expressions displayed in prior studies are ecologically valid and displayed by individuals experiencing actual pain. A recent systematic review of clinical and laboratory studies (Kunz et al., 2019) examined whether acute and chronic pain was associated with consistent movements in specific facial movements, or “action units” (AUs) based on the Facial Action Coding System (FACS; Ekman & Friesen, 1978). Pain was most often associated with brow lowering (AU4), nose wrinkling (AU9, 10), orbital tightening (AU6, 7), and jaw dropping (AU25, 26, 27). While a subset of these units overlap with those that were manipulated in previous studies (Hirsch et al., 2008), no studies have measured how coordinated movements of these regions are interpreted by perceivers and whether sociodemographic features impact assessment. We created computer-generated faces that display coordinated movements across these facial muscles and vary in apparent race and gender. Our goal was to test whether perceivers exhibit sociocultural biases in pain assessment based solely on facial expressions and cues related to sociodemographic features, without further contextual information (i.e., vignettes).

We evaluated whether the intensity of facial movement impacts pain assessments. Manipulating facial expression activation permits insights on the granularity of information with which perceivers can categorize and estimate pain and whether biases in nonverbal pain assessment are stable or dependent on the level of pain expression. In addition, we used a between-groups manipulation to avoid social desirability biases (i.e., response monitoring and demand characteristics). Previous studies have observed effects of social desirability on performance in within-subjects designs when including more than one controversial factor (e.g., Black and White faces; Muslim and Christian vignettes; Walzenbach, 2019) compared to between-subjects designs that only show one factor. Furthermore, decision-making tasks that involve faces from multiple races can engage conflict monitoring (i.e., hyper-awareness of errors) and elicit differential neural activity to Black compared to White faces with the same trial design (Amodio, 2014; Amodio et al., 2008). As previous studies used within-subjects designs to explore the impact of social factors on pain assessment (Hirsch et al., 2008, 2009; Mende-Siedlecki et al., 2019), our goal was to use a between-subjects design to minimize social desirability and conflict monitoring and test whether clinical inequities in pain assessment replicate in experimental contexts.

A second goal of the present study was to measure how pain evaluation relates to emotion recognition. Pain is defined as having both sensory and emotional components and overlaps with emotion in many ways (Gilam et al., 2020). Notably, action units associated with pain (Kunz et al., 2019) overlap with other emotions (see Table 1). For example, AU4 is associated with pain, anger, fear, and sadness, and AU26 is associated with disgust, happiness, pain, and surprise. Although studies have evaluated whether perceivers could distinguish between pain and other basic emotions (Kappesser & Williams 2002; Roy et al., 2015; Simon et al., 2008) or between pain and other negative affective states (e.g., pain and disgust; Antico et al., 2019; Dirupo et al., 2020), participants were presented with only one exemplar or with exemplars from the same race and/or gender; therefore, we do not know if perceivers bias their assessments by target race or the intersection between target race and gender. We aimed to assess not only the relationship between pain and emotion, but also whether the assessment of pain or emotion differs by target race, target gender, or their intersection. Furthermore, studies of emotion recognition indicate that basic emotions are recognized more accurately when assessed by perceivers that share an identity with the target (e.g., same race; Elfenbein & Ambady, 2002), often referred to as an in-group advantage, and perceivers tend to misattribute outgroup faces with stereotyped emotions (e.g., seeing anger in neutral Black faces; Hugenberg & Bodenhausen, 2003) and are less confident and slower to rate outgroup faces (Beaupre & Hess, 2006). Previous studies also indicate that patients who feel similar to their providers experience greater pain relief and lower expectations of pain in patients in simulated (Losin et al., 2017; Necka et al., 2021) and real clinical environments (Nazione et al., 2019; Street et al., 2008). However, we do not know how similarity impacts pain assessments from the perceiver’s position. Therefore, a third aim of our study was to measure how well perceivers can distinguish pain from other emotions on the basis of facial expressions and whether pain recognition and confidence in decisions varies as a function of our sociocultural factors: target sociodemographic features, group status, or similarity.

**Table 1:** Action unit table^a^

*Facial action units*	1	2	4	5	6	7	9	10	12	15	17	20	23	24	25	26	27	43
Anger			x	x							x		x	x				
Disgust						x	x								x	x		
Fear	x	x	x	x		x						x			x			
Happiness					x	x			x						x	x		
Sadness	x		x		x					x	x							
Surprise	x	x		x											x	x		
Pain			x		x	x	x	x							x	x	x	

^a^This table presents action units associated with basic emotions (Keltner et al., 2019) and pain (Kunz et al., 2019). The present study focuses on the contribution of action units associated with pain, which were manipulated collectively rather than individually

Our goals were to examine how participants assess facial expressions of pain and whether pain assessment is biased by sociocultural factors. We also explored how pain assessment relates to emotion recognition. In four studies, participants viewed faces that varied in the magnitude of pain-related facial movement based on empirical studies (Kunz et al., 2019) and in a fifth study participants viewed pain-related expressions as well as basic emotions. In each study, we manipulated the apparent sociodemographic background of the face in a between-subjects design. We controlled the movement of each face, such that movements were matched across sociodemographic subgroups and were restricted to the action units established in Kunz et al., (2019). We used only one combination set of these action units (see Table 1) and made collective changes in their activation (i.e., there was no variability of expression activation within the set of action units) to assess for the effect of facial expression activation on pain assessment outcomes. We hypothesized that increased pain-related facial movement activation would be associated with higher likelihoods of rating pain and higher estimated pain intensity. We also expected these effects to vary by facial identity, such that participants would attribute pain more often and with higher intensity in men and White targets relative to women and Black targets, respectively. We anticipated that these differences would be most pronounced in participants who did not share group membership or similarity with the face target. Finally, we explored the relationship between pain assessment and the assessment of other emotions.

## Method

### Participants

**Fig. 1:**
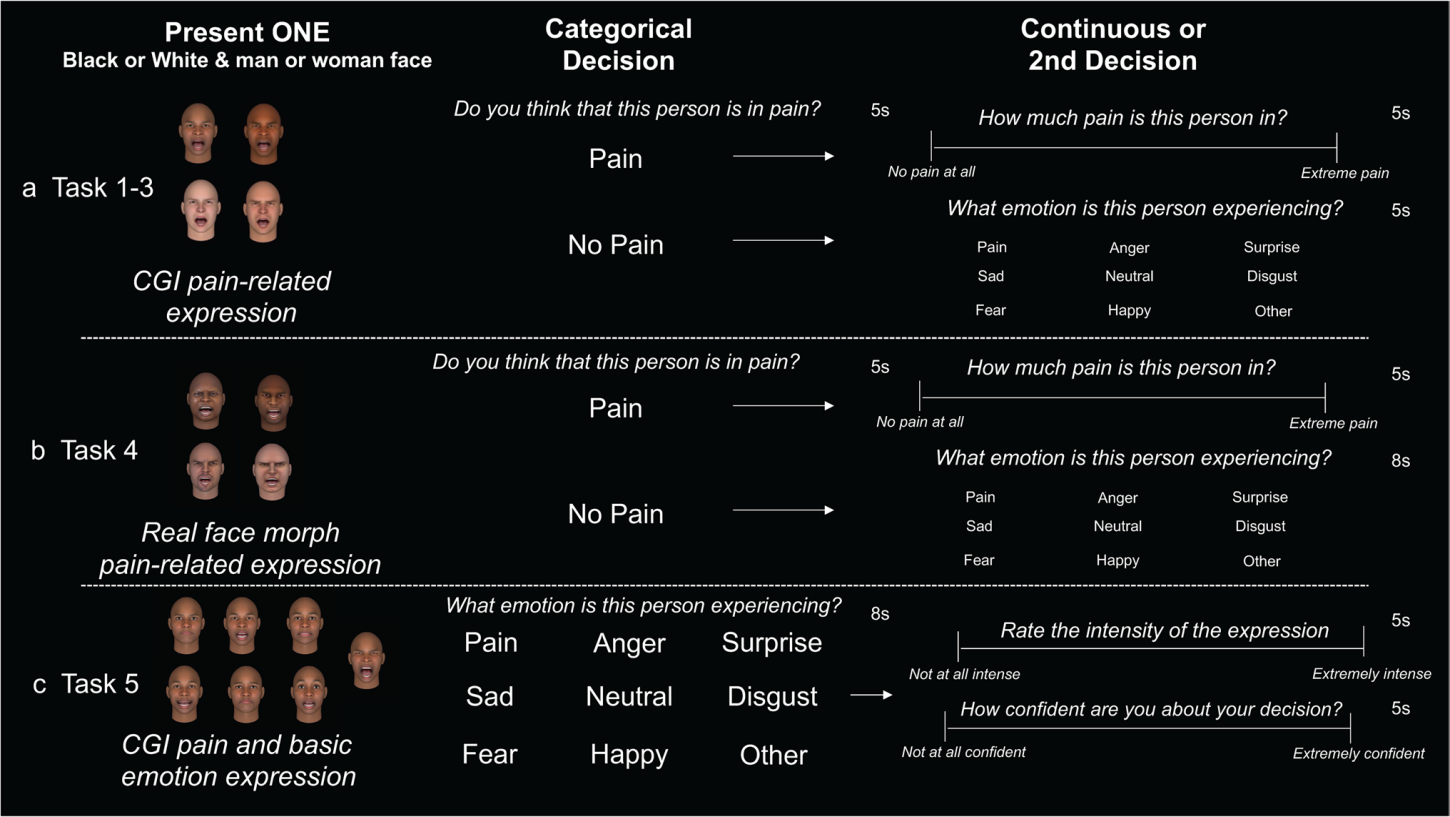
Task schematic. (**a**) Participants were presented with one of four sociodemographic subgroup CGI faces (Black man, Black woman, White man, or White woman). Participants first made a categorical rating of whether they believed the face they were presented was in pain or not. Participants had 5 s to make this rating. If participants chose “No pain,” then they rated whether the individual was experiencing a different emotion or feeling neutral. If participants selected “Pain,” they used a continuous scale to rate how much pain they believed the person was in. (**b**) Participants were presented one of four sociodemographic subgroup faces. Each subgroup included 3 exemplars presented to the participant. Each exemplar was a person that self-identified with their subgroup categories, completed a task with our group, and opted into sharing their facial data. Participants first made a categorical rating of whether they believed the face they were presented was in pain or not. Participants had 5 s to make this rating. If participants chose “No pain,” then they rated whether the individual was experiencing a different emotion or feeling neutral. If participants selected “Pain,” they used a continuous scale to rate how much pain they believed the person was in. (**c**) Participants were presented one of the four sociodemographic faces presented in row A. Each face presented pain, one of the basic emotions (“anger,” “disgust,” “fear,” “happiness,” “sadness,” “surprise”) or a neutral expression. Participants made a categorical rating with each of the category options and other. After a participant selected a category, they rated how intense the expression was and their confidence in their categorical decision. If the participant chose, other, they were prompted to write 1–2 words that best reflected the state the face portrayed

We conducted five online studies between January 2020 and February 2022. Nine hundred sixty-five participants in total (see Table 2 for sample demographics) provided consent as approved under the National Institutes of Health’s Office of Human Subjects Research Protection (18-NCCIH-0622). Enrollment was limited to include participants at least 18 years of age who were currently living in the USA, to ensure participants shared similar societal and cultural contexts. All participants were reached through CloudResearch (formerly TurkPrime; Litman et al., 2017). The task (see Fig. 1 for task schematic) was completed online to increase the diversity and generalizability of our sample (Huff & Tingley, 2015) and recent work has shown CloudResearch’s participant pools exhibit stable demographics, even during the COVID-19 pandemic (Moss et al., 2020). Participants were paid at a rate based on federal minimum wage (i.e., $7.25 per hour). We also implemented the following checks to decrease the potential for any non-human (i.e., bot) or poor responder: participants needed at least a 90% approval rating across a minimum of 100 previously completed online studies, participants could not be on CloudResearch’s “suspicious geolocation” or “bad participants” list (e.g., someone who changes their sociodemographic features across tasks is flagged as a bad participant), and participants could not repeat our task more than once.

**Table 2:** Sample demographics by study

	Study 1	Study 2	Study 3	Study 4	Study 5	Overall
*N*	99	172	270	235	189	965
Sex (male)	55	77 (45%)	111 (41%)	115 (49%)	112 (59%)	470 (48.7%)
Ethnicity: Hispanic/Latinx	15	49 (29%)	72 (26%)	35 (15.8%)	37 (19.5%)	208 (21.5%)
Race: White	81	106 (62%)	157 (58%)	195 (83%)	144 (76%)	683 (70%)
Race: Black	6	17 (10%)	32 (12%)	23 (9.8%)	16 (8.5%)	94 (10%)
Race: Asian	6	21 (12%)	25 (9%)	10 (4.3%)	14 (7.5%)	76 (8%)
Race: Native Hawaiian	2	16 (9%)	24 (9%)	0	0	42 (4.5%)
Race: American Indian or AlaskanNative	0	0	7 (3%)	1 (0.5%)	4 (2%)	12 (1%)
Race: More than one	5	7 (4%)	17 (6%)	4 (1.5%)	9 (5%)	42 (4.5%)
Race: Preferred not to answer	0	5 (3%)	8 (3%)	2 (1%)	2 (1%)	17 (2%)

### Stimuli

All participants completed the experiment via Pavlovia (Peirce & MacAskill, 2018) on either Chrome, Firefox, or Opera web browsers. Participants (“perceivers”) viewed computer generated photos of human faces (“targets”). Previous studies have created pain-related, computer-generated faces using FACEGen (Hirsh et al., 2008) or FACSGen (Antico et al., 2019) softwares*.* We created our images using FaceGen Modeller Pro v3.2 (Singular Inversions, Toronto, CA). Similar to Hirsh and colleagues (2008), we utilized Ekman’s Facial Action Coding System (FACS; Ekman & Friesen, 1978) to define our faces. However, we updated which facial movements to manipulate based on a recent systematic review (Kunz et al., 2019). The targets displayed different magnitudes of facial movement in regions commonly associated with painful stimulation based on FACS including: brow lowering (AU4), jaw dropping (AU25, 26, 27), nose wrinkling (AU9, 10), and orbital tightening (AU6, 7). To increase the ecological validity of the facial movements, we incorporated two additional facial regions, the inner and outer brow (AU1/2) and corner lip pull (AU12), that have been shown to naturally occur with the typically pain evoked facial regions of interest (Fabian Benitez-Quiroz et al., 2016; Zhao et al., 2016). Furthermore, because the intensity of an emotional facial expression can impact how well it is recognized (Hess et al., 1997) and because spontaneous facial expressions of emotions can be less prototypical and recognized less than posed emotions (Krumhuber et al., 2021; Tcherkassof et al., 2007), we varied the intensity of facial movements in our stimuli rather than presenting only maximal expressions. This allowed us to evaluate the association between variations in target facial movement and perceived pain in each analysis.

**Fig. 2:**
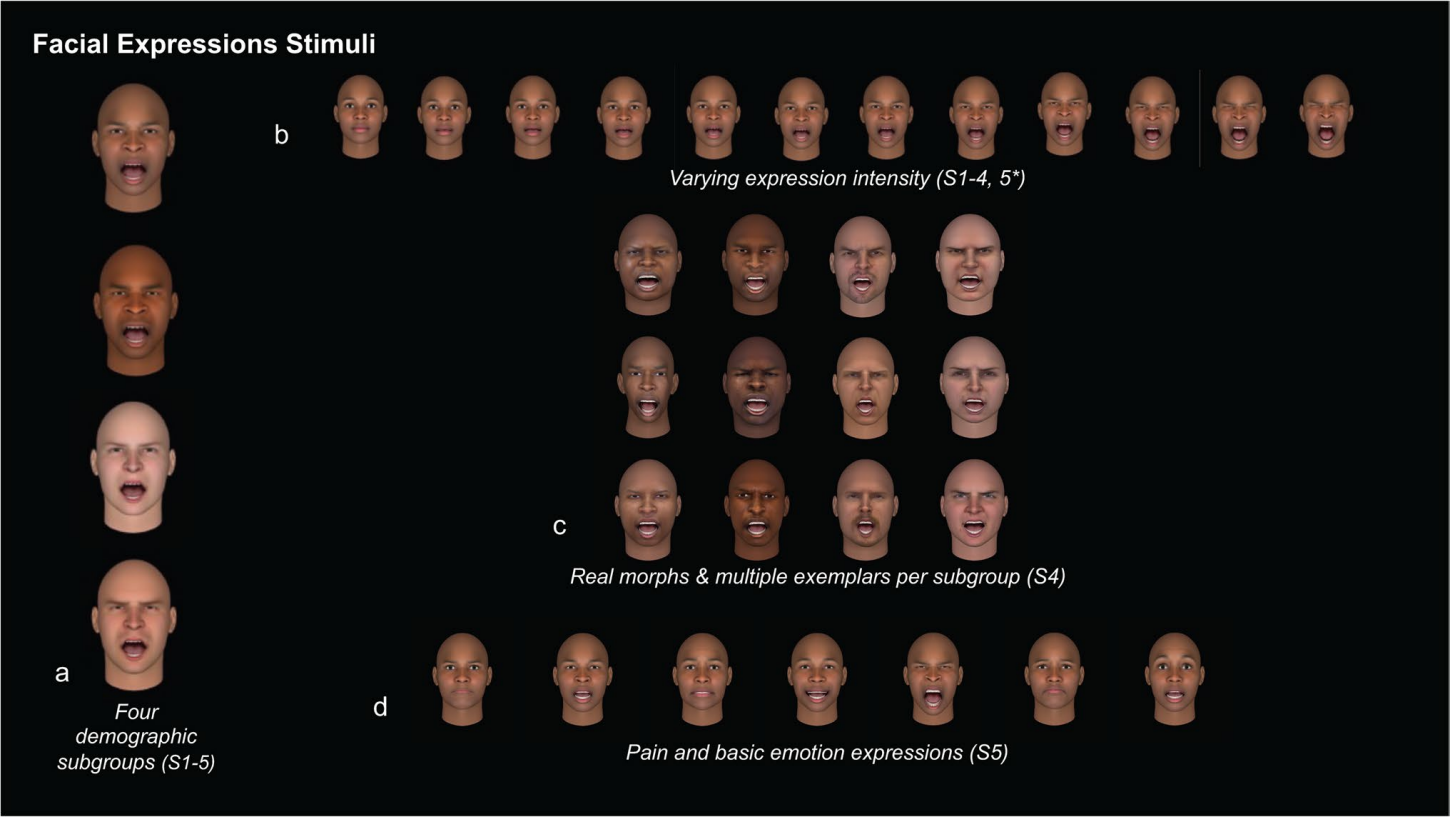
Facial stimuli. (**a**) In each study, participants viewed one of four sociodemographic subgroups (Black man, Black woman, White man, or White woman). The CGI faces presented here were used in Studies 1–3 and 5. (**b**) Stimuli were presented at varying expression intensities. Intensities were increased from 0 to 99% at 9% intervals (i.e., 12 total expressions) for Studies 1–4 and were presented at 0%, 20%, 50%, and 80% in Study 5*. (**c**) In Study 4, individuals who self-identified as a subgroup and consented to sharing their facial data were used as exemplars. Three exemplars were used per subgroup. (**d**) In Study 5, facial expressions of the basic emotions (“anger,” “disgust,” “fear,” “happiness,” “sadness,” “surprise”) and neutral expressions were presented in addition to pain. Each of the additional expressions were also presented at the four intensity levels used in Study 5

In Studies 1–5, expressions were mapped onto four composite faces: White man, White woman, Black man, and Black woman (see Fig. 2). Faces in each study showed equivalent movements across sociodemographic features and took up approximately 30% of the height and 20% of the width of each participant’s screen resolution. The studies were completed online with unfiltered images. This prevented us from computing the visual angle of the stimuli for each of our online participants and from parsing out potential influences of special frequency in our task. However, similar to most studies of pain expression assessment, we presented all stimuli upright and in a front-facing orientation (i.e., we did not apply any vertical or horizontal rotation to the stimuli).

In Studies 1–3, each set of action unit regions were increased collectively from zero (i.e., neutral position) to full activation (99%; i.e., full muscle activation) by 9% for each image, for a total of 12 images per face (i.e., 0%, 9%, 18%, 27%; see Fig. 2).

In Study 4, expressions were mapped onto three exemplars for each of our four sociodemographic groups (i.e., 12 total exemplars). Each exemplar was generated from a photo of a unique individual who participated in an in-person study of pain at NCCIH (ClinicalTrials.gov Identifier: NCT03258580), self-identified with the sociodemographic sub-group and consented to sharing their facial data for use in future studies and for sharing with participants outside of NIH. We used FaceGen Modeller Pro to generate computerized versions of each individual and manipulate the same AUs and intensities as Studies 1–3. Example images are displayed in Fig. 2.

Study 5 incorporated the same four composite faces as Studies 1–3, but in addition to pain-related movements, faces were manipulated to exhibit movements associated with each of the “basic” emotions (“anger,” “disgust,” “fear,” “happiness,” “sadness,” and “surprise”). Action units for the basic emotions were chosen based on the canonical presentations from Keltner et al. (2019). Each expression was presented at 20%, 50%, and 80% activations for a total of 21 images; we also included one neutral image which was presented 3 times to match the number of presentations associated with the other expressions.

### General Procedures

In each study, participants provided consent and then received task instructions (see Supplementary Methods, *Participant Instructions*, for exact language). Each participant was randomly assigned to view only one of the sociodemographic face types (e.g., Black female face) in a between-groups design to decrease the likelihood that participants would think the task was about race or gender bias, which can alter behavior (Amodio et al., 2008, 2006; Devine et al., 2002). Participants performed practice trials with images of their assigned sociodemographic face type to confirm their understanding of the procedures before starting the task.

### Specific Procedures

Studies 1–4: Following the practice, participants completed 36 trials of a pain assessment task in which twelve facial configurations (see Fig. 2) were each presented three times across the task. In Studies 1–3, participants saw a single identity 36 times, whereas in Study 4, participants saw three exemplars from the same sociodemographic group each 12 times (36 total trials). On each trial, the participant saw a target face and made a binary choice to report whether the target they viewed was in pain or not (see Fig. 1a). The target face was shown simultaneously with the options of “pain” and “no pain” and perceivers had 5 s to make their rating. If the perceiver rated the target face as displaying “pain,” they then had 5 s to rate the target’s pain intensity. Pain intensity was rated on a continuous scale anchored on the lower end with “No pain at all” and “Extreme pain” at the upper end (see Fig. 1a). For convenience, the scale was treated as a 0–7 continuous visual analogue scale in analyses, but there were no numbers displayed during the task (see Fig. 1a). The target face remained on the screen while the perceiver made their judgment. If a participant did not rate in the allotted time (5 s), the trial was not included in analyses. If the perceiver selected “no pain,” they then reported which emotion the target expressed: options included “anger,” “disgust,” “fear,” “happy,” “sad,” “surprise,” “other,” and “neutral” (see Fig. 1a). The emotion categories were presented in the same order each trial in Studies 1–3 and randomized in Study 4. Participants were allotted a few extra seconds to respond in Study 4 (8 s total), to account for the randomization.

Following 36 trials of the pain assessment task, the perceiver answered a free-response question probing what parts of the face they used to make their pain rating decisions. This question was used to probe for bots in Study 2 and Study 3; however, most participants left this question blank (Study 2 = 58% of participants; Study 3 = 63% of participants). Participants who left this question blank were still included in analyses. To improve our ability to identify suspicious responses, we used a multiple-choice option for Study 4, which asked which type of stimuli subjects were being asked to rate. All participants answered this question correctly.

We also collected ratings of perceived similarity. Perceivers rated how similar they believed the target face they viewed was to themselves on a continuous scale anchored from “Not at all similar” to “Extremely similar.” Participants were not primed to rate similarity based on any particular criteria. In Study 4, participants provided similarity ratings for each of the three facial identities that they viewed. Finally, participants self-reported their age, gender, race, and ethnicity.

#### Study 5

Study 5 had a similar structure to Studies 1–4 but included six emotions (anger, disgust, fear, happy, sad, and surprise) in addition to pain. Each expression type was presented at three activation levels (20%, 50%, and 80% activation) across the task. We also included three neutral expressions (0% activation). We again used a single target face for each sociodemographic group (i.e., the same exemplars as Studies 1–3).

Following the practice trials, participants completed 24 trials in which participants first categorized the target’s expression and then provided ratings of intensity and confidence (see Fig. 1c). The emotion categories were randomized each trial, and participants had 8 s to make each decision. As in Studies 1–4, intensity ratings were collected using a continuous visual analogue scale anchored with “Not at all intense” at the lower end and “Extremely intense” at the upper end, which was treated as a continuous 0–7 continuous scale in analyses, although there were no numbers displayed during the task. The target face remained on the screen, while the perceiver made their intensity judgment (5 s to make judgment). Following the intensity rating, participants were shown the emotion category they had chosen and rated confidence in their categorization on a continuous scale with “Not at all confident” at the lower end and “Extremely confident” at the upper end (5 s to make judgment). On trials in which participants selected “other” rather than a specific expression, they did not provide intensity or confidence ratings and instead had 10 s to provide a free text response to the prompt, “Please type 1–2 words of what you believe this face is expressing.” Trials in which participants failed to respond were not included in analyses. Similar to Study 4, we included a multiple-choice attention check, which asked which type of stimuli subjects were being asked to rate. All participants answered this question correctly.

### Statistics and Analyses

#### Preregistration and Power Analyses

Sample size for study 1 was selected by running a power analysis in G*Power (Erdfelder et al., 1996) to estimate the number of participants needed to run a fully powered study to observe bias in pain categorization. We used the effect size reported for the experiments with computer-generated faces by Mende-Siedlecki and colleagues (2019; *F* = 0.8) with alpha set at 0.05 and power set at 80%. Studies 2 through 4 were preregistered at AsPredicted (Study 2, https://aspredicted.org/q22q7.pdf; Study 3, https://aspredicted.org/za5t5.pdf; Study 4: https://aspredicted.org/PF1_GQN), and powered based on the effects from the preceding studies with the same alpha and power parameters. We powered Study 5 based on a significant Target Gender (between) by Emotion (within) interaction, with a weighted effect size f(V) of 0.3246, from Studies 1–3 (Study 4 and Study 5 occurred simultaneously). We preregistered and included our power analysis for the study at AsPredicted (https://aspredicted.org/VPK_ZWK). In each of our studies, we did not remove outliers from our analyses. To take advantage of our multiple studies and to streamline our results, we deviated from our preregistration by using meta-analyses to assess for reliability and consistency of our effects (described below) compared to our initial plan to use Bayesian statistics within each study. Preregistered secondary analyses (e.g., reaction time, post-task qualitative questions) may be analyzed separately in future work.

#### Data Processing

We downloaded each perceiver’s data as CSV files from Pavlovia and imported the data to R v3.6.1 (R Core, Vienna, Austria). We evaluated catch questions to identify suspicious responses or bots. No participants were excluded from any study. Trials on which subjects failed to respond (*M*_*Study 1*_ = 0.13 trials; *M *_*Study 2*_ = 0.54 trials; *M *_*Study 3*_ = 0.86 trials; *M *_*Study 4*_ = 0.37; *M *_*Study 5*_ = 0.24) were excluded from analyses. Based on the correspondence between a perceiver’s self-identified race and gender and the sociodemographic category of the target they viewed, we computed two group membership measures. We created a group membership by gender variable, in which an individual was designated as either ingroup (perceiver shared the same gender as the target) or outgroup (perceiver did not share the same gender as the target). Similarly, we created a group membership by race variable with the same designations of ingroup or outgroup status.

#### Statistical Analyses: Studies 1–4

Studies 1–4 were analyzed using the same procedures. Because each study used the same basic design with subtle variations (e.g., stimuli, sample size, period of data collection), we used meta-analyses to make inferences across the studies. Complete results from individual studies are reported in Supplementary Tables (S1–S13).

#### Multilevel Models

We used multilevel models to assess the effect of facial expression activation on pain categorization and pain intensity estimates and whether target race, gender, group membership, or similarity had an impact on these effects. We evaluated maximal models (Barr et al., 2013) using both the lmer and glmer functions in the lme4 package in R (Bates et al., 2015). Although our similarity measure allowed for more nuance compared to group membership (i.e., a perceiver can have varying levels of similarity towards targets that may be equivalent by group membership), our factors for similarity and group membership had high multicollinearity; therefore, we evaluated each factor in separate models.

We used logistic multilevel models in each study to assess the odds a trial was rated as painful or not-painful based on expression intensity (i.e., do greater facial expression activations increase the odds of rating a trial as painful) and whether any of our sociocultural measures impacted this relation. If a participant did not have at least three trials in which they observed pain and three trials in which they observed no-pain, then they were excluded from the multilevel logistic models (final *n*_Study1_ = 87, *n*_Study2_ = 160, *n*_Study3_ = 257, *n*_study4_ = 226). We also used multilevel linear models to assess the effect of facial muscle movement (“ExpressionActivation”) on intensity ratings on trials rated as painful in each study and to evaluate whether sociocultural measures impacted this relationship. For each multilevel model, we verified homoscedasticity of our residuals. We also report the full model formulas in the *Supplementary Methods* and model betas and significances for each of our predictors in the *Supplementary Tables*.

#### Meta-analyses

We originally preregistered Bayesian analyses for our studies; however, as Studies 1–4 shared the same overall structure and asked the same question, we used meta-analyses to assess the reliability of our effects and focus on inferences that were consistent across samples and subtle task variations. Meta-analyses were implemented using the “metagen” function within the “meta” package for R (Balduzzi et al., 2019). Standardized betas and standard errors were taken from each multilevel model to compute meta-analyses across Studies 1–4 in R for each factor. Betas, 95% confidence intervals, and associated *p* values computed across Studies 1–4 are reported for each factor and represented in random forest plots with the forest function within the meta package in R.

#### Statistical Analyses: Study 5

We used repeated measures ANOVAs to estimate perceived intensity and reported confidence in our fifth study. ANOVAs were implemented with the aov_car function within the afex package (Singmann et al., 2015) in R. As we had limited number of trials, we ran separate models for our within-subjects factors (i.e., emotion type and facial activation). We ran a 2 (gender: Women, Men; between) × 2 (race: White, Black; between) × 8 (emotion: Anger, Disgust, Fear, Happiness, Neutral, Pain, Sadness, Surprise; within) repeated measures ANOVA and a 2 (gender: Women, Men; between) × 2 (race: White, Black; between) × 3 (facial activation: low [20%], medium [50%], high [80%] expression; within) repeated measures ANOVA. We used the emmeans package in R (Lenth et al., 2019) to run Tukey HSD tests on any factor that was significant in our model. All post hoc comparisons are included in Supplementary Tables (S14–S17). We analyzed separate models for our two dependent measures, perceived intensity and confidence.

We also computed a confusion matrix of emotion recognition as a function of category to assess how often individuals’ perceptions matched the putative emotion category of the expression. Finally, we computed hit rates using frameworks built by Wagner (1993) and Kappesser and Williams (2002). Simple hit rates (*h*_1_) were the proportion of correctly identified emotion categories, sensitivity hit rates (*h*_2_) were the number of correct responses compared to the number of times the category was chosen, and unbiased hit rate (*h*_u_) was the product of these two hit rates.

## Results

### Perceivers Attribute Pain to Faces Displaying Pain-Related Movements

We first examined the proportion of trials that participants perceived the target in pain, relative to no pain. On average perceivers rated pain on approximately half of the trials (total trials = 36) in each study (*M*_Study1_ = 51%, SD_Study1_ = 17%; *M*_Study2_ = 54%, SD_Study2_ = 21%; *M*_Study3_ = 54%, SD_Study3_ = 20%; *M*_Study4_ = 48.8%, SD_Study4_ = 17%). Analyses of responses on no-pain trials are reported below (“The relationship between pain and other emotions depends on experimental context”).

**Fig. 3:**
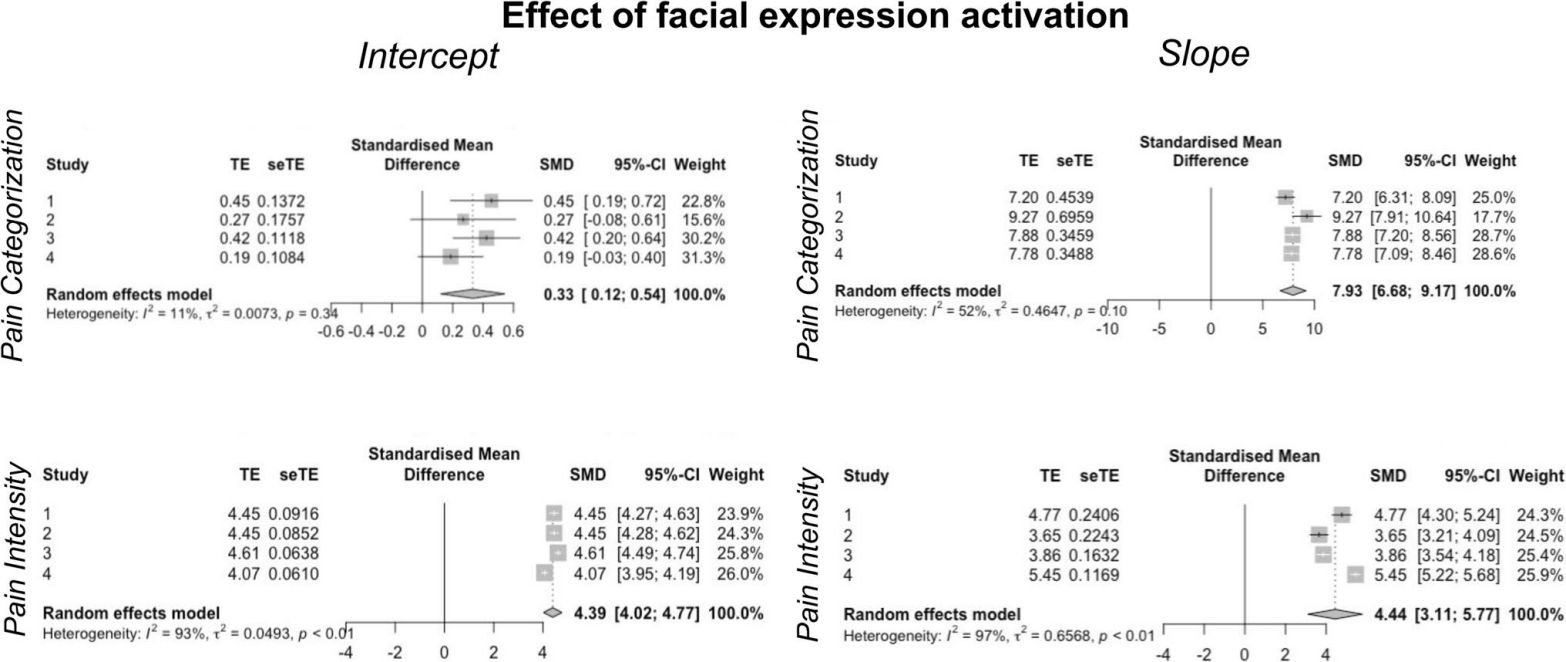
Meta-analyses of the association between facial expression activation and pain categorization and intensity. We conducted a meta-analysis on intercepts and slopes from logistic and linear models across Studies 1–4 using the metagen function in R (Balduzzi et al., 2019). We observed a significant intercept for both pain categorization (p = 0.02) and intensity (p < 0.001), and we observed a significant effect of facial activation on slope for both pain categorization (p < 0.001) and intensity (p = 0.002), suggesting that perceivers are able to use facial activation information to identify pain and its intensity in targets

We used multilevel logistic regressions (implemented in “glmer”) to analyze whether the magnitude of pain-related facial expression activation impacted pain recognition (i.e., pain vs. no pain judgments). We first examined the first-level (i.e., within-subjects) associations between target facial movement and the likelihood of labeling a target as being in pain. Meta-analysis of intercepts from Studies 1–4 revealed a significant positive intercept (SMD = 0.33, 95% CI [0.12, 0.54], *p* = 0.02; Fig. 3), such that perceivers rated the average pain-related facial expression activation as painful. Furthermore, across Studies 1–4, we observed a significant positive slope for the association between facial expression activation and pain categorization (SMD = 7.93, 95% CI [6.68, 9.17], *p* < 0.001; Fig. 3), such that perceivers were more likely to categorize an image as painful at more active facial expressions in all studies. Full model results for the meta-analyses are reported in Supplementary Table S1 and each individual study’s outcomes are reported in Supplementary Tables (Study 1, S2–S4; Study 2, S5–S7; Study 3, S8–S10; Study 4, S11–S13).

### Pain-Related Facial Muscle Activation Is Positively Associated with Perceived Pain Intensity

We used multilevel linear models to assess the association between the magnitude of pain-related facial muscle activation and estimated pain intensity on trials rated as painful. Meta-analysis of intercepts across Studies 1–4 revealed a significant positive intercept (SMD = 4.39, 95% CI [4.02, 4.77], *p* < 0.001; Fig. 3), such that perceivers rated the average pain-related facial expression activation at 63% between anchors of “no pain” and “extreme pain” (i.e., 4.39 on our 0–7 scale for scoring). Furthermore, across Studies 1–4, we observed a significant positive association between facial expression activation and perceived pain intensity (SMD = 4.44, 95% CI [3.11, 5.77], *p* = 0.002), such that larger facial movements were rated as more intense.

### Sociocultural Factors Do Not Reliably Affect Pain Categorization or Perceived Pain Intensity

**Fig. 4:**
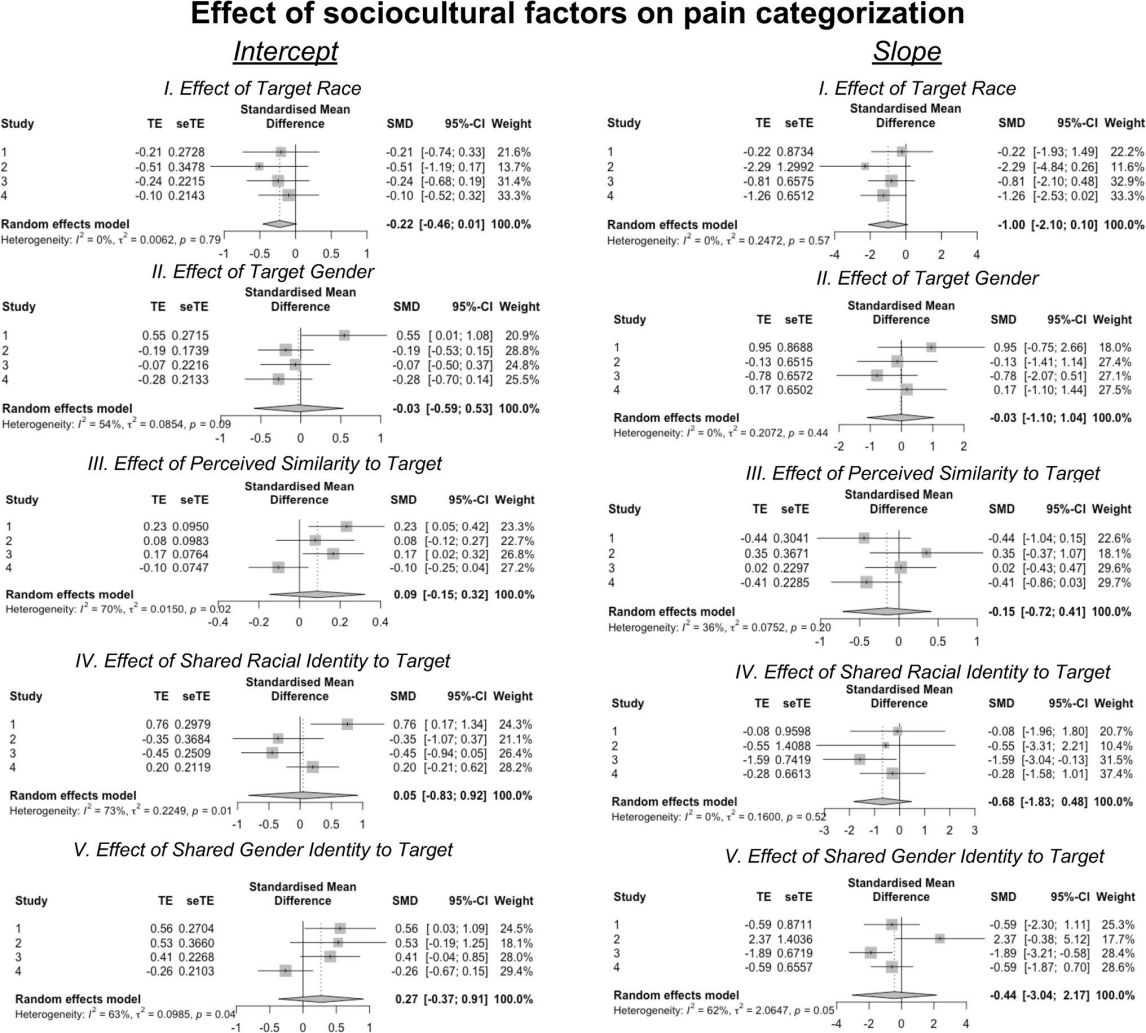
Meta-analyses of effects of sociodemographic factors on likelihood of pain assessment. We conducted a meta-analysis of the effects of sociocultural factors (target demographics, perceived similarity, and group membership) on intercepts (left column) and slopes (right column) from logistic models across Studies 1–4 using the metagen function in R (Balduzzi et al., 2019). There were no consistent influences of sociocultural factors across studies. For full model outcomes, please see Supplementary Table S1

Our key questions were whether target gender, race, group-membership, or perceived similarity impacted the effect of facial movement activation on pain categorization or perceived pain. To evaluate these questions, we examined interactions between first-level (within-subjects) and second-level (i.e., between-subjects) factors in both logistic and linear models. Figure 4 depicts results of meta-analyses of logistic models evaluating the association between sociocultural factors and the likelihood of categorizing a trial as painful. Meta-analysis of intercepts across Studies 1–4 revealed no effects of sociodemographic factors on the likelihood of rating pain at the average facial expression (all *p*’s > 0.05). Furthermore, across Studies 1–4, we did not observe any effects of sociodemographic features on the association between facial muscle movement and the pain categorization (all *p*’s > 0.05). Full model results are located in Supplementary Table S1.

**Fig. 5:**
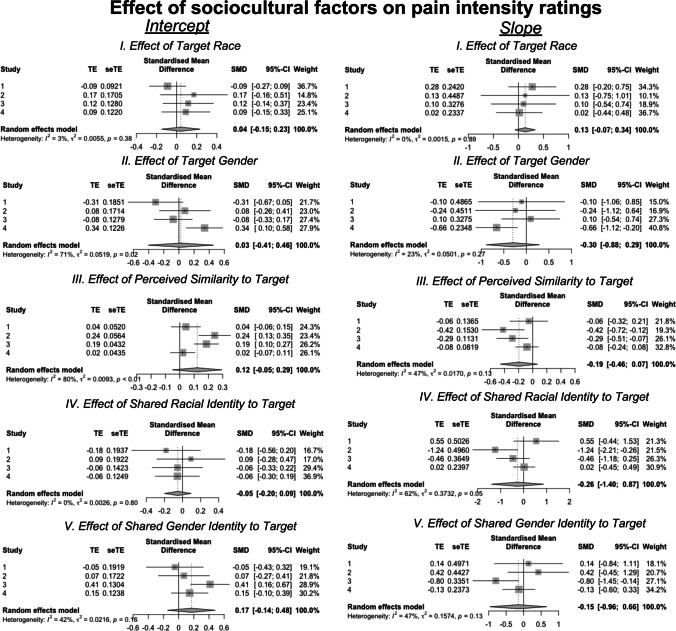
Meta-analyses of effects of sociodemographic factors on pain intensity estimates. We conducted a meta-analysis of the effects of sociocultural factors (target demographics, perceived similarity, and group membership) on intercepts (left column) and slopes (right column) from linear models across Studies 1–4 using with the metagen function in R (Balduzzi et al., 2019). There were no consistent influences of sociocultural factors across our studies. For individual study results, please see “Supplementary Information”

Figure 5 depicts meta-analyses of the relationships between sociodemographic factors and the association between facial movements and perceived pain intensity on trials estimated as painful (i.e., linear models). Meta-analyses across Studies 1–4 revealed no consistent relationship between sociodemographic factors and the intercept (i.e., pain intensity at average facial expression; all *p*’s > 0.05) or the slope (association between facial activation and perceived pain; all *p*’s > 0.05).

### The Relationship Between Pain and Other Emotions Depends on Experimental Context

**Fig. 6:**
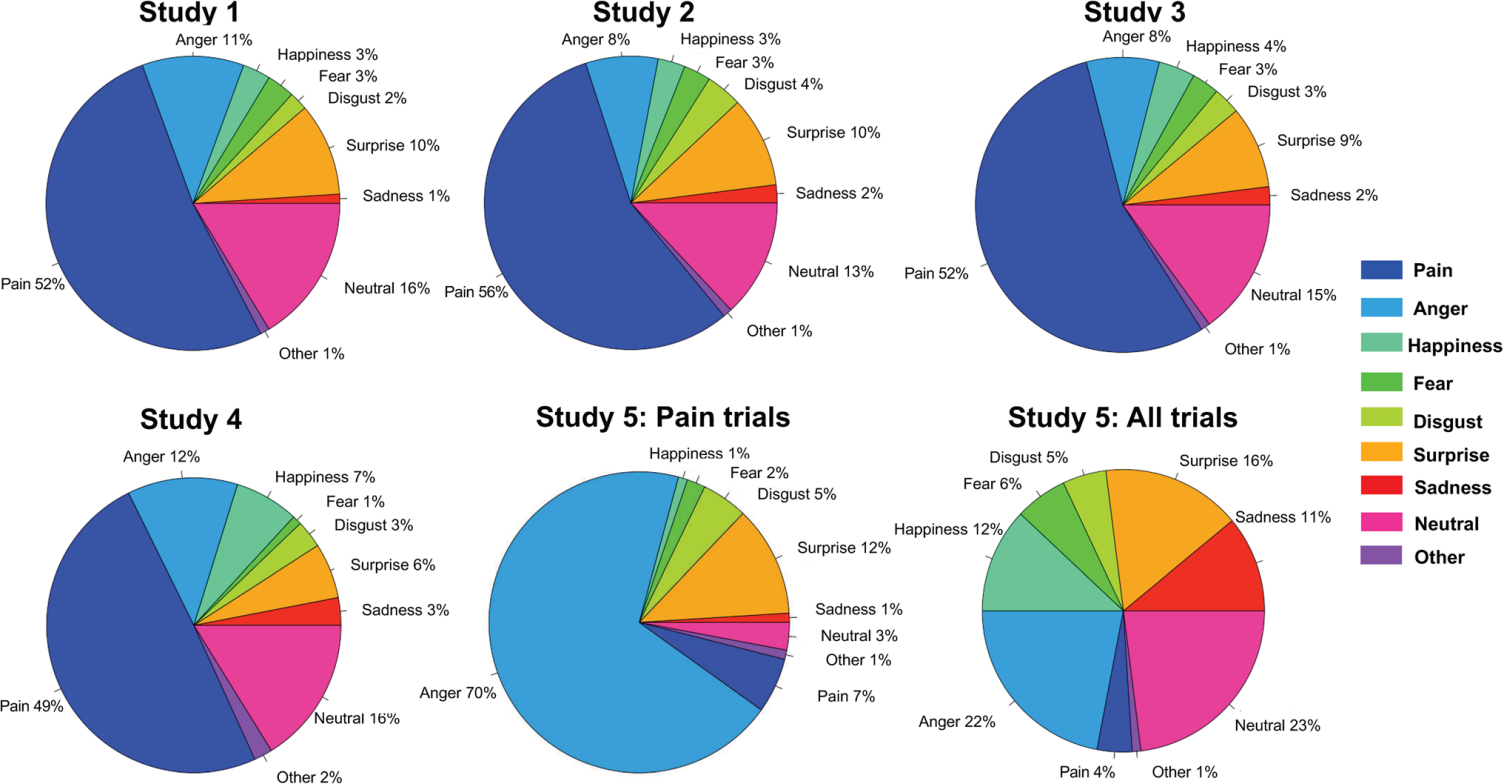
Pie charts for mean emotion recognition rates across all subjects. In Studies 1–4, which each included a pain categorization question, perceivers attributed pain to the faces on the majority of trials, whereas Study 5, which included all of the basic emotions in the initial categorization question, perceivers rarely chose pain. We present Study 5 in two pie charts, as this study also included several non-pain emotion expressions: “Study 5: Pain trials” includes emotion attributions on trials that presented pain-related expressions and “Study 5: All trials” includes all trials during the task

The results reported above focus on Studies 1–4, in which targets displayed varying intensities of movement in facial muscles previously associated with pain (Kunz et al., 2019). Importantly, on trials that were rated as not painful, perceivers categorized which other emotion they observed. In Studies 1–4, the emotions that comprised the greatest percentage of trials were “Neutral” (*M*_Study1_ = 14.5%, SD_Study1_ = 9.8%; *M*_Study2_ = 12.4%, SD_Study2_ = 9.7%; *M*_Study3_ = 14.4%, SD_Study3_ = 10.1%; *M*_Study4_ = 16.3%, SD_Study4_ = 7.9%), “Surprise” (*M*_Study1_ = 11.9%, SD_Study1_ = 9.6%; *M*_Study2_ = 9.7%, SD_Study2_ = 9.4%; *M*_Study3_ = 8.9%; SD_Study3_ = 8.7%; *M*_Study4_ = 6.1%; SD_Study4_ = 5.4%), and “Anger” (*M*_Study1_ = 10.1%, SD_Study1_ = 10.3%; *M*_Study2_ = 8.7%, SD_Study2_ = 11.4%; *M*_Study3_ = 7.8%, SD_Study3_ = 11.2%; *M*_Study4_ = 12.1%, SD_Study4_ = 12.3%). For complete results, see Fig. 6.

**Fig. 7:**
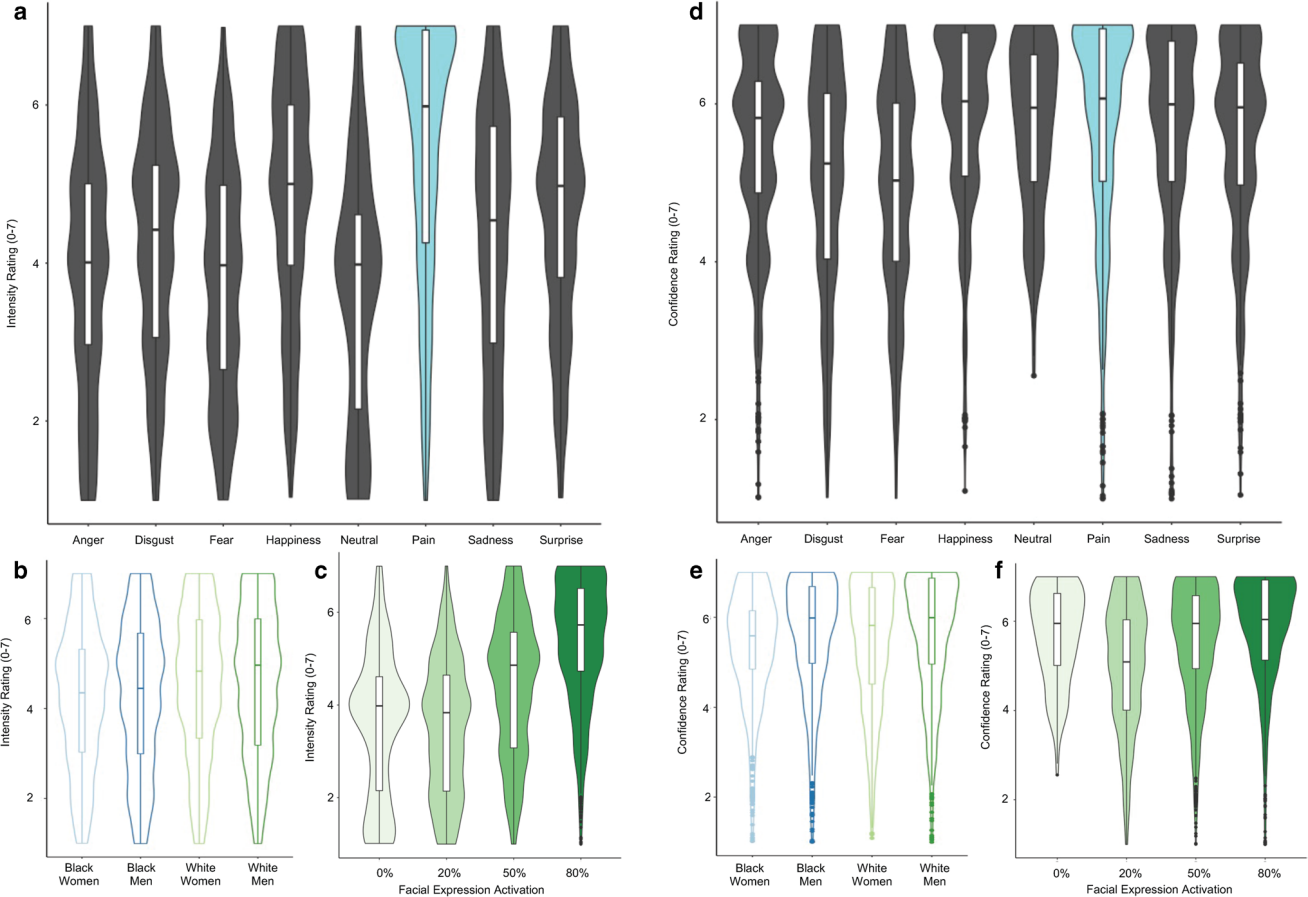
Repeated measures ANOVAs. (**a**) We observed a main effect of target emotion on perceived intensity ratings (p < 0.001). Intensity ratings were highest when pain expressions were shown (blue hued violin plot). (**b**) We observed a main effect of target race on perceived intensity (p = 0.03), such that White targets (in green) were attributed with more intensity than Black targets (in blue). (**c**) We observed a main effect of target facial expression activation on perceived intensity (p < 0.001), such that more intensity was attributed at higher facial expression activations. (**d**) We observed a main effect of target emotion on perceived confidence (p < 0.001). Pain (in blue) was among emotions that were rated with the most confidence. (**e**) We observed a main effect of target gender on confidence, such that women targets (in the lighter shades) were rated at lower confidence compared to men targets (in the darker shades; p = 0.05). (**f**) We observed a main effect of target facial expression activation on perceived intensity, such that more intensity was attributed at higher facial expression activations and at the lowest category (for neutral expressions; p < 0.001)

Because Studies 1–4 included only pain-related expressions and may have primed participants to judge pain, we conducted a fifth study in which pain was equally likely to be expressed as other emotions. We used repeated-measures ANOVAs to analyze effects of our experimental manipulations on both perceived intensity and perceiver confidence. Results are reported in Table 3. In a model that included target emotion, target race, and target gender, we observed main effects of target emotion, F(4, 783) = 134.5, *p* < 0.001, n_g_^2^ = 0.23 and target race, F(1, 185) = 4.89, *p* = 0.03, *n*_g_^2^ = 0.02 on intensity ratings (see Fig.7a–c). Post hoc analyses with Tukey’s HSD tests indicated that participants attributed higher intensity ratings to stimuli that depicted pain (*M* = 5.45, *SD* = 1.62) compared to other emotion categories (*M*_range_ = [3.6–4.84]*, SD*_range_ = [1.43–1.73]), as depicted in Fig. 7a. For a full consideration of post hoc comparisons in perceived intensity by target emotion, see Supplemental Table S14. Perceivers also reported greater intensity in White targets (*M* = 4.50, *SD* = 1.65) compared to Black targets (*M* = 4.23, *SD* = 1.65; see Fig. 7b). There was no effect of Target Gender on perceived intensity (*p* > 0.6), and there were no significant interactions between any factors (all *p*’s > 0.1). When we included target facial expression activation (target activation) as a factor rather than target emotion, we observed a main effect of target activation, F(2, 308) = 260.0, *p* < 0.001, n_g_^2^ = 0.33, such that perceivers attributed higher intensity ratings to more expressive faces (*M*_*80%*_ = 5.40; *SD*_*80%*_ = 1.35) compared to less expressive faces (*M*_*20%*_ = 3.50; *SD*_*20%*_ = 1.43; see Fig. 7c). In the rmANOVA with target activation, there was no longer an effect of target race (*p* = 0.06), and there were no other main effects or interactions present (all *p*’s > 0.05). For complete results, see Table 3 and post hoc comparisons in Supplemental Table S15.

**Table 3:** Study 5 rmANOVAs

*DV*	*IV*	*Effect*	*df*	*MSE*	*F*	*ges*	*p*
*Intensity rating*	*Target expression activation*	Target gender	1, 185	3.13	0.13	< .001	0.72
		Target race	1, 185	3.13	3.67	0.013	0.057
		Target gender*target race	1, 185	3.13	0.08	< .001	0.771
		Target activation	1.66, 307.86	1.02	259.96	0.331	< .001
		Target gender*target activation	1.66, 307.86	1.02	0.16	< .001	0.815
		Target race*target activation	1.66, 307.86	1.02	2.02	0.004	0.143
		Target gender*target race*target activation	1.66, 307.86	1.02	0.26	< .001	0.734
	*Target emotion*	Target gender	1, 185	5.32	0.16	< .001	0.689
		Target race	1, 185	5.32	4.89	0.016	0.028
		Target gender*target race	1, 185	5.32	0.04	< .001	0.85
		Target emotion	4.23, 783.23	0.85	134.5	0.227	< .001
		Target gender*target emotion	4.23, 783.23	0.85	1.16	0.003	0.329
		Target race*target emotion	4.23, 783.23	0.85	1.51	0.003	0.195
		Target gender*target race*target emotion	4.23, 783.23	0.85	1.13	0.002	0.34
*Confidence Ratings*	*Target expression activation*	Target gender	1, 185	2.25	4.64	0.018	0.032
		Target race	1, 185	2.25	0.01	< .001	0.921
		Target gender*target race	1, 185	2.25	0.01	< .001	0.91
		Target activation	2.31, 427.53	0.38	84.91	0.113	< .001
		Target gender*target activation	2.31, 427.53	0.38	1.39	0.002	0.249
		Target race*target activation	2.31, 427.53	0.38	2.55	0.004	0.071
		Target gender*target race*target activation	2.31, 427.53	0.38	1.02	0.002	0.368
	*Target emotion*	Target gender	1, 185	4.4	4	0.013	0.047
		Target race	1, 185	4.4	0.04	< .001	0.843
		Target gender*target race	1, 185	4.4	0	< .001	0.981
		Target emotion	5.99, 1107.93	0.47	46.6	0.089	< .001
		Target gender*target emotion	5.99, 1107.93	0.47	1.12	0.002	0.349
		Target race*target emotion	5.99, 1107.93	0.47	1.65	0.003	0.131
		Target gender*target race*target emotion	5.99, 1107.93	0.47	1.11	0.002	0.356

When we used similar repeated measures ANOVAs to examine effects of sociodemographic factors and target emotion category on confidence ratings (see Fig. 7d–f), we observed main effects of target emotion, F(6, 1108) = 46.6, *p* < 0.001, n_g_^2^ = 0.09 and target gender, F(1, 185) = 4.0, *p* = 0.05, n_g_^2^ = 0.01. There were no effects of target race (*p* > 0.8) nor any interactions between factors (all *p*’s > 0.07). Post hoc comparisons indicated that perceivers were most confident when rating pain (*M* = 5.78*, SD* = 1.35*)*, neutral (*M* = 5.71*, SD* = 1.26), and happiness (*M* = 5.86*, SD* = 1.14), relative to all other emotion categories (*M*_range_ = [4.98–5.68]*, SD*_range_ = [1.25–1.42]; see Fig. 7d). Perceivers also reported higher confidence in assessments of targets depicting men (*M* = 5.61, *SD* = 1.32) compared to targets depicting women (*M* = 5.39, *SD* = 1.3). For a full consideration of post hoc comparisons in perceived confidence by emotion category, see Supplemental Table S16. When we replaced target emotion category with target activation to measure the effects of facial expression intensity on confidence, we observed a main effect of target activation, F(2, 428) = 84.9, *p* < 0.001, n_g_^2^ = 0.11, such that confidence varied as a function of facial expression intensity (see Fig. 7f). The effect of target gender remained significant in this model (*p* = 0.03), and no other main effects or interactions were present (all *p*’s > 0.05). For full comparison of confidence by target activation see Table 3, and for post hoc comparisons see Supplemental Table S17.

**Fig. 8:**
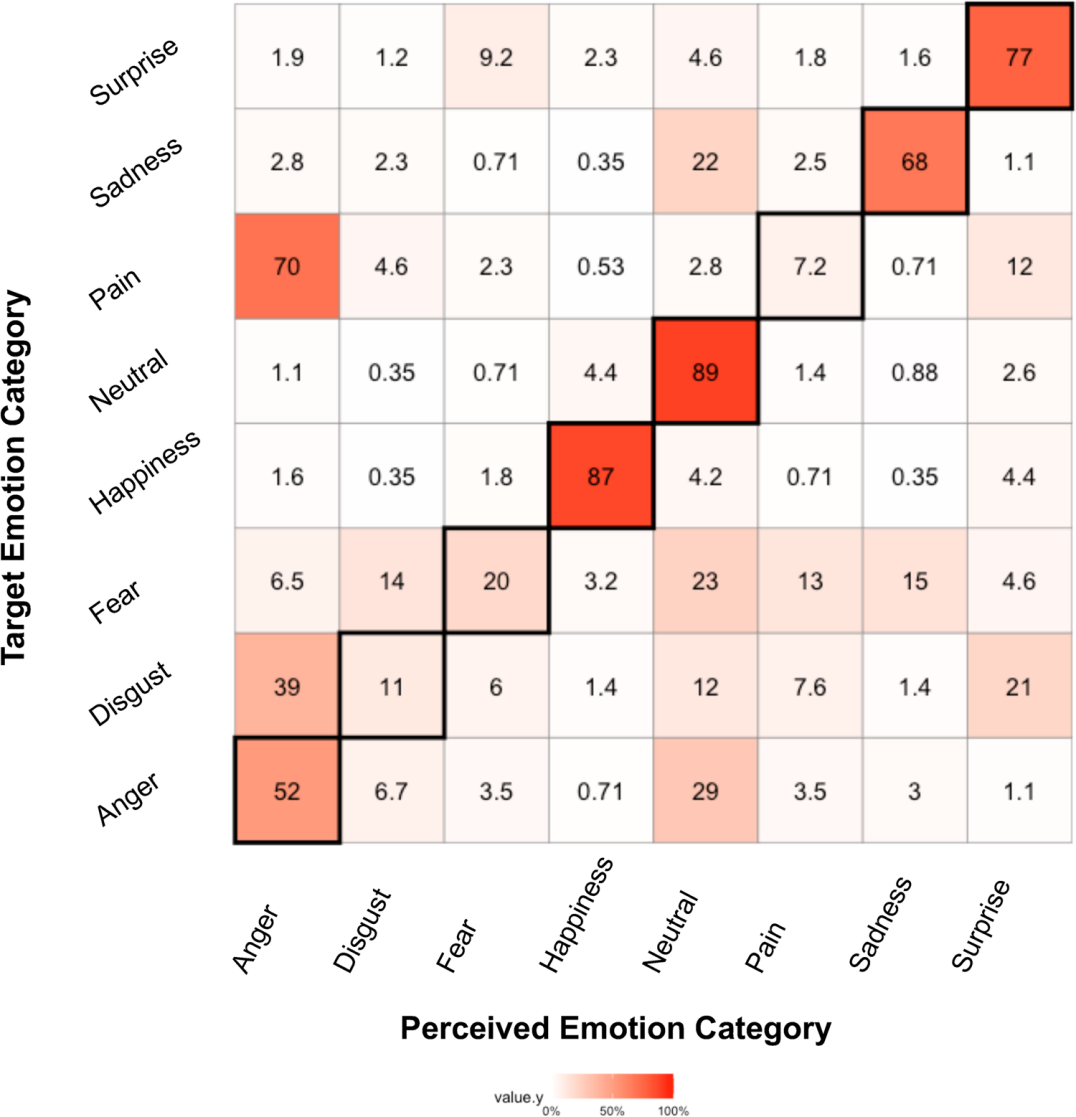
Confusion matrix of emotion recognition. On the x-axis are the attributions that perceivers made when viewing each image, “Perceived Emotion Category,” and the y-axis are emotions as they were created and displayed, “Target Emotion Category.” The darker red colors signify a better mapping between canonical representation and perception, whereas the lighter colors signify less mapping and more confusion. Numbers in each box represent the percentage a category was selected. These numbers always equate to 100% by row and may differ from 100% by column if they were selected more often (greater than 100%) or less often (less than 100%) by perceivers

Although pain expressions elicited high intensity ratings and reported confidence, a confusion matrix reveals that pain stimuli were one of the most confused emotion categories (see Fig. 8). Pain stimuli were only perceived as pain expressions 7.2% of the time and were confused for anger expression 70% of the time (compared to other emotions when restricted to their presentations: anger 52%, disgust 11%, fear 20%, happiness 87%, neutral 89%, sadness 68%, and surprise 77%). Although we did not evaluate the effect of facial expression activation on expression confusion statistically due to power limitations, we have included confusion matrices limited to each of the activation levels in “Supplementary information” (see Supplementary Figure S1). Visual inspection suggests pain-related movements are perceived as anger mainly at the 50% and 80% facial expression activations. Across all emotions, there were more misattributions at lower activation levels (see Supplementary Figure S1), but pain was never recognized as pain above 10% of trials regardless of intensity. Similarly, pain expression stimuli had the least accuracy (i.e., lowest unbiased hit rate) of all emotion categories (see Table 4).

**Table 4:** Hit rate estimates by emotion categories

*Photo*	*Hit rates*		
	H_1_ (%)	H_2_(%)	H_U_ (%)
*Chance*	1/9 = 11.1%	1/8 = 12.5%	11.1% * 12.5% = 1.4
*Anger*	52.2	29.8	15.5
*Disgust*	10.8	26.8	2.9
*Fear*	20.5	45.8	9.4
*Happiness*	86.6	87.1	75.4
*Neutral*	88.5	47.3	41.9
*Pain*	7.2	18.8	1.4
*Sadness*	68.1	74.8	50.9
*Surprise*	77.4	62.3	48.2

Simple hit rates (*h*_1_) were the proportion of correctly identified emotion categories, sensitivity hit rates (*h*_2_) were the number of correct responses compared to the number of times the category was chosen, and unbiased hit rate (*h*_u_) was the product of these two hit rates

## Discussion

We investigated how pain-related facial movements influence pain assessment and whether perceivers’ ratings depend on target or perceiver sociodemographic features. We observed that ecologically valid movements are recognized as pain, sociocultural factors did not consistently affect perceivers’ recognition and estimation of pain, and pain expressions were less recognizable than other emotion categories.

### Pain-Related Facial Activations Are Positively Associated with Pain Recognition and Intensity Estimation

Stimuli from previous literature on facial expressions of pain recognition typically consist of cartoons (Wong et al., 2005), actors portraying pain expressions (Mende-Siedlecki et al., 2019), or facial images chosen from a large set by other perceivers (Chen et al., 2018). These faces are important for understanding what types of pain expressions people expect to see and portray; and although some studies have verified that these expectations matched real expressions (e.g., Chen et al., 2018), it is unknown whether socially marginalized patients exhibit such expressions in the clinic. We based our stimulus expressions on a recent systematic review (Kunz et al., 2019) of facial movements (brow lowering, nose wrinkling, jaw dropping, and orbital tightening) and observed that our perceivers were still capable of recognizing and estimating pain in these facial movements.

It is important to note that in our first four studies, perceivers recognized pain in our faces on a majority of trials, but that recognition depended on facial movement activation. Images with less than 50% facial expression activation had lower odds of being perceived as in pain. This is consistent with studies on basic emotions, which find emotions are more easily decoded at more intense facial expressions (Hess et al., 1997). This suggests that perceivers may have a hard time recognizing pain when a person exhibits subtle or suppressed pain-related facial movements. Once participants labeled a target as painful, we observed that facial activation was positively associated with estimated pain intensity. Furthermore, like other modalities and pain decisions that elicit higher confidence with more sensory information (Dildine et al., 2020), perceivers reported higher levels of confidence in their ratings on trials with more facial activation. Our results provide empirical evidence that individuals can rate pain-related nonverbal responses in a graded manner and can engage in metacognitive processes during these decisions.

### Target Gender, Race, Group Status, and Perceived Similarity Had Inconsistent Effects on Pain Assessment but Influenced Intensity and Confidence in Emotion Judgments

Although we hypothesized differences in pain outcomes as a function of social factors, we did not observe consistent pain assessment biases as a function of sociocultural measures. Although these findings stand in contrast to the important clinical literature on pain disparities from which we based our hypotheses (i.e., that pain tends to be underassessed in Black patients in the clinic; Green et al., 2003), we note that some clinical studies have failed to observe sociocultural influences on pain. For instance, 43% of studies reviewed in one literature review of pain and ethnicity (Cintron & Morrison, 2006) did not report disparities in pain assessment and management. We also note that data collection for at least one sub-study (Study 2) was collected during a period of social unrest in the United States (Buchanan et al., 2020), when Black pain and suffering were broadcast widely (Dave et al., 2020). The timing and social context might have primed participants differently compared to studies conducted before the social unrest.

Interestingly, although we did not observe consistent influences of race or gender on pain assessment, we did observe an influence of target race on perceived intensity of emotion, and an influence of target gender on confidence in emotion judgements. These effects were present across emotion categories and were not specific to any particular emotion. Prior studies indicate that sociodemographic features typically do not influence emotion categorization, but do elicit biases in evaluations or behaviors associated with the categories (e.g., reaction time; Bijlstra et al., 2010). Sociodemographic features and how they relate to the perceiver can alter the interpretation of emotions and influence one’s responses to the emotion (Lazerus et al., 2016; Weisbuch & Ambady, 2008). However, most prior research focuses on differences in implicit metrics (e.g., reaction time). Our results suggest that sociodemographic features can also alter explicit features of emotion recognition, namely perceived intensity and confidence. We build on previous work comparing pain and emotion decisions (Blais et al., 2019; Dirupo et al., 2020; Kappesser & Williams 2002; Roy et al., 2015; Simon et al., 2008; Wang et al., 2021) by observing that intensity ratings were biased by target race and confidence ratings were influenced by target gender. Future studies of pain assessment should explore whether stereotypes have different impacts on these different types of judgments.

### Perceivers Rarely Identify Pain and Confuse It for Other Emotions when Provided with Many Emotion Categories

Although pain expressions were associated with high levels of confidence and elicited intensity ratings that tracked facial movement, pain was consistently confused for other emotions in our final study. Each emotion, including pain, was presented on 12.5% of trials (3 trials total per emotion). On average, participants only labeled putatively pain-related movements as pain 7.2% of the time (*M*_trials_ = 1.72), whereas movements related to happiness were recognized as happiness on 87% of presentations, anger was recognized on 52% of presentations and surprise, a supposedly ambiguous emotion (Neta et al., 2020) was recognized on 77% of trials. Instead, pain related movements were most likely to be perceived as anger (70% of trials). Interestingly, the facial action units most frequently associated with anger (AUs: 4, 5, 17, 23, 24) overlap less with pain (AUs: 4, 6, 7, 9, 10, 25, 26, 27; Kunz et al, 2019) than other emotions (e.g., AUs associated with disgust: 7, 9, 25, 26). Indeed, in previous studies examining pain and emotion decisions, pain has been most often confused as disgust (Kappesser & Williams 2002) as one would predict based on AU overlap; however, the same study also observed anger expressions were the second most likely emotion (after pain) to be labelled as pain. Pain and anger are both high in negative valence and arousal and thus overlap in the affective circumplex (Russell, 1980). Furthermore, three of the five action units that commonly appear during anger are on the lower part of the face (AU17 chin raiser, AU20 lip stretcher, and AU23 lip tightener). Although these specific action units were not part of our pain-related faces, we did include the movement of multiple action units around the mouth (AU25/26/27). This is dissimilar to previous studies of pain-related facial movement which did not include these action units or any action units around the mouth (Prkachin, 1992). In the present study, we also may have observed lower levels of recognition because there were several negative emotion categories (similar to lower levels of recognition for negative affective states observed in previous studies of pain and emotion; Roy et al., 2015), whereas we only presented one positive emotion (happiness), and the recognition rate was highest for happiness. However, other negative emotions were associated with higher recognition rates than pain, and thus there may be something uniquely ambiguous about pain-related expressions, particularly when pain is presented alongside other emotions. These results suggest that pain-related facial movements without priming (i.e., “pain” or “no pain” used in Studies 1–4) are difficult to perceive as pain and suggest pain-related facial movements may be hard to decode when the stimulus is devoid of other cues (e.g., body movement) as observed in previous studies (Aviezer et al., 2012). This stands in stark contrast to Studies 1–4, in which most trials were perceived as painful. In these initial studies, perceivers were primed with an initial pain or no pain categorization question; however, participants were still able to select other emotions. Thus, future studies should continue work on the relationship between pain and other emotional expressions (Chen et al., 2018) as well as how context and priming may shape the assessment of pain and emotion.

### Limitations

Our study adds to the literature by measuring the impact of facial movements that were associated with acute or chronic pain in previous literature (Kunz et al., 2019). Yet because most studies of facial responses to pain have been restricted to White and Western samples, and because our selection of facial movements was based on these studies, we do not know whether the facial movements that we presented generalize across sociocultural groups. Future research should measure pain assessment in images of diverse individuals experiencing actual pain, which would improve ecological validity and help us determine if current action units associated with pain are generalizable across diverse samples. However, studies of real individuals in pain must account for variations in pain experience and expression across individuals, which is why computer-generated stimuli offer some advantages. Our stimuli also lacked hair, clothing, and other potential social status indicators. Although this leads to a more controlled comparison of gender and has been incorporated in previous research (Oosterhof & Todorov, 2008), it may also decrease potential interactions between status or context and gender that may amplify bias (Freeman et al., 2011, Hirsch et al., 2008). Incorporating additional cues may be important in future work, as only a few studies have considered whether culture influences pain expressions (Chen et al., 2018). The interaction between culture, context, and pain-related expressions is important to understand, as expressiveness might vary across cultures and or as a function of patient-provider interactions, as individuals may try to counteract negative stereotypes (e.g., medication seeking) when seeking medical care. Future research should examine how sociocultural factors may impact a patient’s nonverbal pain behaviors and how these behaviors impact providers’ decisions. It is possible that biases in the clinic may be driven in part by the healthcare context and social interaction.

Our stimuli presented uniform movement intensities across action units, which prevented us from isolating potential facial regions and movements that may be under or overutilized in assessing pain. The eight action units used in our study, combined into four distinct regions by Kunz and colleagues, were the most common and only regions consistently active above baseline in the review by Kunz et el. (2019); however, pain-related expressions are accompanied by individual differences in action-unit activation (Kunz et al., 2019) and these regions of action-units are not always active during pain (Kunz et al., 2014). Future research should independently manipulate not only the four sub-regions, but all eight action units, to measure each action units’ unique contributions to pain assessment and test for potential interactions.

Finally, we ran our study online with a general sample population that acted as a proxy for medical providers. Future studies should investigate the role of nonverbal assessments and potential influences of sociocultural factors in medical providers, who have been shown to assess false beliefs about Black patients’ pain (Hoffman et al., 2016). Nonetheless, laypeople also endorse stereotypes and false beliefs, and thus, we believe our data can provide important insights. It is important to note that our sample was skewed, with a majority of participants identifying as White. Similar skews also exist in the make-up of medical professionals (Ly & Jena, 2021). This leads to skew in our ingroup/outgroup race variable, which overrepresents White targets as ingroup race and Black targets as outgroup race. Although we did not observe different results when restricting ingroup and outgroup analyses to Black perceivers, future studies should use specific approaches to obtain a more balanced sample. In addition, perceivers that did not identify as either Black or White were always categorized as outgroup race. Including more exemplars and identities beyond the typically used Black and White categories would increase the generalizability of our studies. Furthermore, future studies would benefit from delineating factors like similarity that may arise from multiple and individualized criteria (e.g., similarity scores may be driven by a belief in shared personality and/or a belief in shared appearances). Finally, future studies should include potential moderators, like perceiver’s racial bias endorsement, time spent in the USA, and community diversity to help identify why sociocultural biases in pain assessment may arise.

## Conclusions

Our results suggest perceivers can recognize and assess nonverbal pain-related facial movements. Although perceivers do require a certain level of facial movement to consistently recognize pain, perceivers are able to utilize pain-related facial movements to recognize and estimate pain intensity. Furthermore, increased levels of facial expression activation increased recognition of emotions and confidence in perceiver judgments. However, without priming or cues perceivers rarely identify pain-related facial expressions and confuse it for other emotions. Finally, we observed inconsistent effects of target gender, race, group status, and perceived similarity on pain assessments similar to prior experimental paradigms investigating pain assessment biases. Overall, more work is needed to understand how assessment biases in the clinic form to move toward interventions and more equitable healthcare and future studies should further investigate the relationship between pain assessment, emotion recognition, and the psychosocial context surrounding pain and its treatment.

## Supplementary Information

Supplementary file1 (DOCX 3447 KB)

Supplementary file2 (PNG 3775 KB)

## Data Availability

All data and scripts are available upon request. https://osf.io/xjvae/?view_only=bb82d8d5ffb74f6fb216cdaba6530612.
