# Supplementary material for: How Pain-Related Facial Expressions Are Evaluated in Relation to Gender, Race, and Emotion
Source: Affect Sci. 2023 Mar 3;4(2):350–69. doi: 10.1007/s42761-023-00181-6 (PMC9982800; doi:10.1007/s42761-023-00181-6)
Supplement: Supplementary file 1 — Supplementary file1 (DOCX 3447 KB) [file 42761_2023_181_MOESM1_ESM.docx]

Supplementary Information to accompany,

*“How pain-related facial expressions are evaluated in relation to gender, race, and emotion”*

Troy C. Dildine, Carolyn M. Amir, Julie Parsons & Lauren Y. Atlas

Supplementary Methods

*Participant Instructions.* A participant began the study by reading over the consent and information about the study. If they agreed to participate, they then went through instructions for the task. The participant was prompted that they would view images of people in pain and must make ratings:

“You will see images of people and indicate whether or not you think the person is in pain.”

“You will see a person’s face. Try to make a quick decision about whether or not this person is in pain.”

“Click on ‘pain’ to indicate if a face is in pain and on ‘no pain’ to indicate a face is not in pain. If you select ‘pain,’ you will use the mouse to indicate how much pain you think the person is in. If you select ‘no pain,’ you will be asked whether the person portrayed a different emotion. These emotions include: Happy, Sad, Anger, Fear, Disgust, Surprise, Neutral, or Other.”

After participants were told what type of images they would be viewing and what ratings they would be making, we had them complete practice trials with one of the faces they would view during the task and the text below.

“Do you think that this person is in pain? Try clicking on ‘pain’ to indicate that the person is in pain.”

“Good. Now try indicating how much pain the person was in on the scale.”

“There may also be trials in which you do not think the person exhibits pain. Try clicking on ‘no pain’ to indicate this person is not in pain.”

“If you choose no pain you will asked to identify another emotion. What emotion is this person experiencing?”

After completing the practice trials, participants continued to the main task. Participants were given our contact information if they had any questions about the task, and they were notified that they should be mindful as to how they contacted us to ensure privacy.

*Model Formulas.* We used logistic multilevel models in each study to assess the odds a trial was rated as painful or not-painful based on expression intensity. The following models were evaluated using the program ‘glmer’:

1. Target race and gender

**ln**

**PainRating_ij_  = (**g**_00_ +** g**_10_ExpressionActivation_ij_ +** g**_01_Race_ij_ +** g**_01_Gender_j_ +**

**No-Pain Rating_ij_** g**_01_Race*Gender_j +_** g**_01_Gender*ExpressionActivation_j +_** g**_01_Race* ExpressionActivation_j +_** g**_01_Race*Gender*ExpressionActivation_j +_  ) + (*u*_0j_ + *u*_0_ExpressionActivation_ij_) + r_ij_**

1. Similarity

**ln**

**PainRating_ij_  = (**g**_00_ +** g**_10_ExpressionActivation_ij_ _+_** g**_01_Similarity_ij_ +**

**No-Pain Rating_ij_** g**_01_Similarity* ExpressionActivation_j ij_ ) + (*u*_0j_ +**

***u*_0_ExpressionActivation_ij_) + r_ij_**

1. Group membership

**ln**

**PainRating_ij_  = (**g**_00_ +** g**_10_ExpressionActivation_ij_ +** g**_01_GroupRace_ij_ +**

**No-Pain Rating_ij_** g**01GroupGenderj +** g**01GroupRace*GroupGender_j +_** g**01GroupGender*ExpressionActivation_j +_** g**01GroupRace* ExpressionActivation_j +_** g**01GroupRace* GroupGender* ExpressionActivation_j +_  ) + (*u*_0j_ + *u*_0_ExpressionActivation_ij_) + r_ij_**

We also used multilevel linear models to assess the effect of facial muscle movement (“ExpressionActivation”) on intensity ratings on trials rated as painful in each study. The following models were implemented using the program ‘lmer’:

1. Target race and gender

**PainIntensity_ij_  = (**g**_00_ +** g**_10_ExpressionActivation_ij_ +** g**_01_Race_ij_ +** g**_01_Gender_j_ +**

g**_01_Race*Gender_j +_** g**_01_Gender*ExpressionActivation_j + +_** g**_01_Race* ExpressionActivation_j +_** g**_01_Race*Gender*ExpressionActivation_j_ ) + (*u*_0j_ + *u*_0_ExpressionActivation_ij_) + r_ij_**

1. Perceived similarity

**PainIntensity_ij_  = (**g**_00_ +** g**_10_ExpressionActivation_ij_ +** g**_01_Similarity_ij_ +**

g**_01_Similarity* ExpressionActivation_j ij_ ) + (*u*_0j_ + *u*_0_ExpressionActivation_ij_) + r_ij_**

1. Group membership

**PainIntensity_ij_  = (**g**_00_ +** g**_10_ExpressionActivation_ij_ +** g**01GroupRaceij +**

g**01GroupGenderj +** g**01GroupRace*GroupGender_j +_** g**01GroupGender*ExpressionActivation_j +_** g**01GroupRace* ExpressionActivation_j +_** g**01GroupRace* GroupGender* ExpressionActivation_j_) + (*u*_0j_ + *u*_0_ExpressionActivation_ij_) + r_ij_**

Supplementary Figure

*S1. Confusion matrix of emotion recognition at each activation level (20%, 50% and 80%)*

*
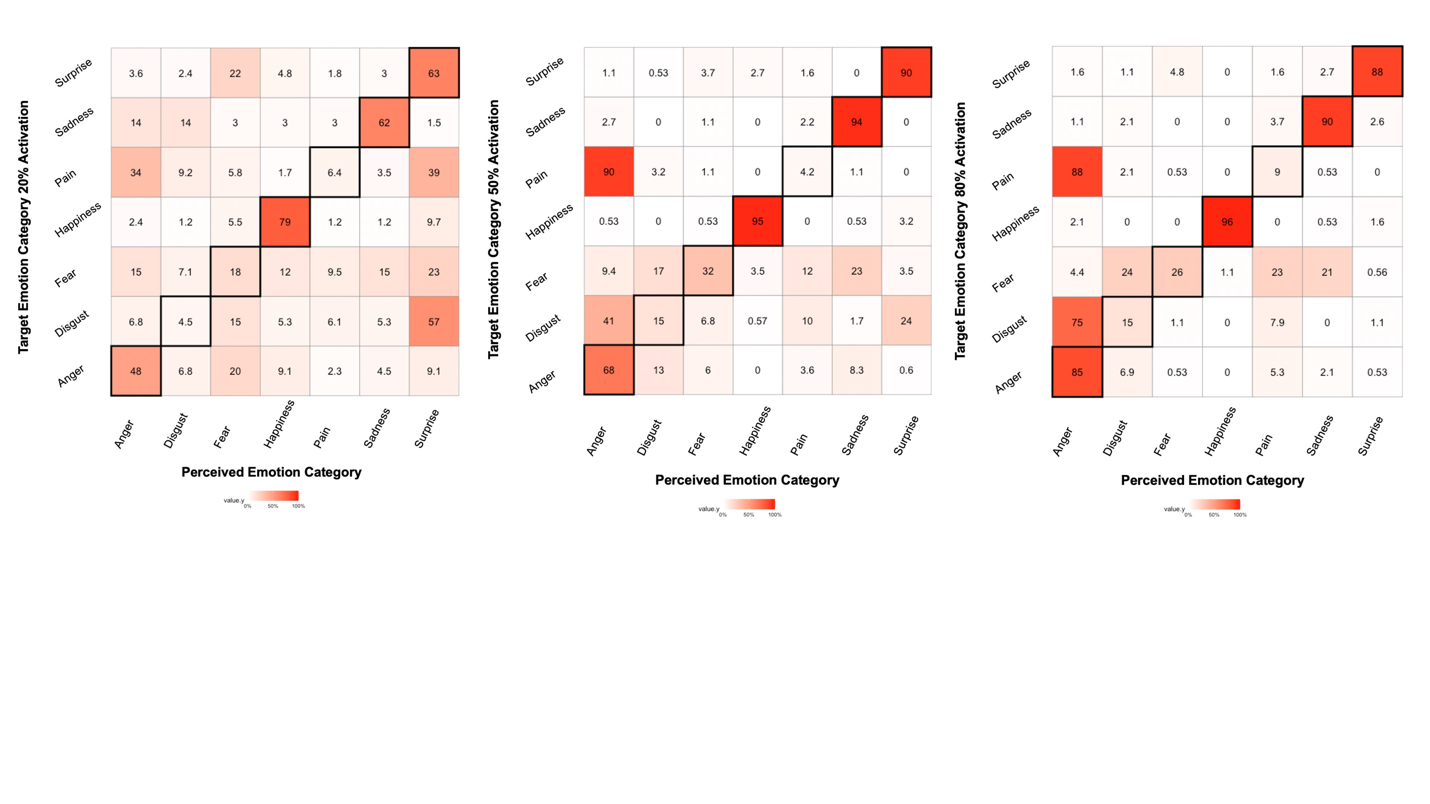
*

Figure S1. *Confusion matrix of emotion recognition at each activation level (20%, 50%, 80%).* On the x-axis are the attributions that perceivers made when viewing each image and the y-axis are the emotions that we tried to build based on canonical representations. The darker red colors signify a better mapping between canonical representation and perception, whereas the lighter colors signify less mapping and more confusion. Rows represent how frequently each category was selected for a target emotion category (all numbers in the column should sum to 100%). Columns represent the percent of trials for which each target category was selected as the perceived emotion. A combined value of 100% across each row denotes the emotion category being chosen as often as it is presented, whereas values above or below 100% indicate that category was chosen more or less frequently than it was presented. Neutral expressions were set at 0% and therefore are not a part of the activation based confusion matrices.

Supplementary Tables

Table S1. Results from meta-analyses of Studies 1-4

|  |  | **Intercept** | |  | |  | |  | | **Slope** | |  | |  | |  | |
| --- | --- | --- | --- | --- | --- | --- | --- | --- | --- | --- | --- | --- | --- | --- | --- | --- | --- |
|  |  | ***SMD*** | ***CI*** | | ***t*** | | ***p*** | | ***SMD*** | | ***CI*** | | ***t*** | | ***p*** | |  |
|  | Facial Expression | 0.33 | [.12, .54] | | 5.00 | | 0.02 | | 7.93 | | [6.68, 9.17] | | 20.27 | | <.001 | |  |
|  | Target Race | -0.22 | [-.46, .01] | | -2.99 | | 0.06 | | -0.99 | | [-2.1, .10] | | -2.88 | | 0.06 | |  |
| Pain Categorization | Target Gender | -0.03 | [-.59, .53] | | -0.15 | | 0.89 | | -0.03 | | [-1.1, 1.04] | | -0.09 | | 0.93 | |  |
|  | Perceived Similarity | 0.12 | [-.05, .29] | | 1.19 | | 0.32 | | -0.19 | | [-.46, .07] | | -0.86 | | 0.45 | |  |
|  | Group Race | 0.05 | [-.83, .92] | | 0.18 | | 0.87 | | -0.68 | | [-1.83, .48] | | -1.87 | | 0.16 | |  |
|  | Group Gender | 0.27 | [-.37, .91] | | 1.34 | | 0.27 | | -0.44 | | [-3.04, 2.17] | | -0.53 | | 0.63 | |  |
|  | Facial Expression | 4.39 | [4.01, 4.77] | | 37.14 | | <.001 | | 4.44 | | [3.11, 5.77] | | 10.61 | | 0.002 | |  |
|  | Target Race | 0.04 | [-.15, .23] | | 0.70 | | 0.53 | | 0.13 | | [-.07, .34] | | 2.10 | | 0.13 | |  |
| Pain  Intensity | Target Gender | 0.03 | [-.41, .46] | | 0.18 | | 0.87 | | -0.30 | | [-.88, .29] | | -1.62 | | 0.20 | |  |
|  | Perceived Similarity | 0.12 | [-.05, .29] | | 2.25 | | 0.11 | | -0.19 | | [-.46, .07] | | -2.36 | | 0.10 | |  |
|  | Group Race | -0.05 | [-.2, .09] | | -1.13 | | 0.34 | | -0.26 | | [-1.4, .87] | | -0.74 | | 0.51 | |  |
|  | Group Gender | 0.17 | [-.14, .48] | | 1.72 | | 0.18 | | -0.15 | | [-.96, .66] | | -0.59 | | 0.60 | |  |

Table S2. Study 1: Assessing the role of target demographics on pain outcomes

|  | **Variable** | **β** | **SE** | **z/t*** | ***p*** |
| --- | --- | --- | --- | --- | --- |
| *Logistic*  *MLM* | *Intercept* | 0.455 | 0.137 | 3.314 | 0.001 |
|  | *Facial Activation* | 7.202 | 0.454 | 15.866 | < .001 |
|  | *Target Race* | 0.545 | 0.272 | 2.009 | 0.045 |
|  | *Target Gender* | -0.206 | 0.273 | -0.754 | 0.451 |
|  | *Facial Activation *Target Race* | 0.955 | 0.869 | 1.099 | 0.272 |
|  | *Facial Activation *Target Gender* | -0.219 | 0.873 | -0.251 | 0.802 |
|  | *Facial Activation *TargetRace*Target Gender* | -1.070 | 0.546 | -1.959 | 0.050 |
| *Linear*  *MLM* | *Intercept* | 4.451 | 0.092 | 48.575 | < .001 |
|  | *Facial Activation* | 4.772 | 0.241 | 19.836 | < .001 |
|  | *Target Race* | -0.308 | 0.185 | -1.664 | 0.1 |
|  | *Target Gender* | -0.090 | 0.092 | -0.983 | 0.329 |
|  | *Facial Activation *Target Race* | -0.105 | 0.487 | -0.215 | 0.83 |
|  | *Facial Activation *Target Gender* | 0.277 | 0.242 | 1.143 | 0.256 |
|  | *Facial Activation *TargetRace*Target Gender* | -0.090 | 0.187 | -0.481 | 0.632 |

*Z scores are presented from logistic multilevel models and t scores are presented for linear multilevel models. Logistic models were computed using glmer and linear models were computed using lmer in R. The current model assessed perceiver biases by target demographics.

Table S3. Study 1: Assessing the role of group membership on pain outcomes

|  | **Variable** | **β** | **SE** | **z/t** | ***p*** |
| --- | --- | --- | --- | --- | --- |
| *Logistic*  *MLM* | *Intercept* | 0.456 | 0.137 | 3.340 | < .001 |
|  | *Facial Activation* | 7.234 | 0.455 | 15.914 | < .001 |
|  | *Group Race* | 0.756 | 0.298 | 2.536 | 0.011 |
|  | *Group Gender* | 0.557 | 0.270 | 2.059 | 0.039 |
|  | *Facial Activation *Group Race* | -0.078 | 0.960 | -0.081 | 0.936 |
|  | *Facial Activation *Group Gender* | -0.595 | 0.871 | -0.683 | 0.495 |
|  | *Facial Activation *GroupRace* GroupGender* | -0.286 | 0.594 | -0.481 | 0.630 |
| *Linear*  *MLM* | *Intercept* | 4.448 | 0.095 | 46.921 | < .001 |
|  | *Facial Activation* | 4.845 | 0.246 | 19.718 | < .001 |
|  | *Group Race* | -0.182 | 0.194 | -0.940 | 0.350 |
|  | *Group Gender* | -0.051 | 0.192 | -0.267 | 0.790 |
|  | *Facial Activation *Group Race* | 0.548 | 0.503 | 1.090 | 0.279 |
|  | *Facial Activation *Group Gender* | 0.139 | 0.497 | 0.280 | 0.780 |
|  | *Facial Activation *GroupRace* GroupGender* | 0.014 | 0.397 | 0.035 | 0.972 |

Table S4. Study 1: Assessing the role of similarity on pain outcomes

|  | **Variable** | **β** | **SE** | **z/t** | ***p*** |
| --- | --- | --- | --- | --- | --- |
| *Logistic*  *MLM* | *Intercept* | 0.454 | 0.139 | 3.276 | 0.001 |
|  | *Facial Activation* | 7.199 | 0.453 | 15.904 | < .001 |
|  | *Similarity* | 0.232 | 0.095 | 2.439 | 0.015 |
|  | *Facial Activation * Similarity* | -0.444 | 0.304 | -1.461 | 0.144 |
| *Linear*  *MLM* | *Intercept* | 4.451 | 0.092 | 48.270 | < .001 |
|  | *Facial Activation* | 4.812 | 0.241 | 19.945 | < .001 |
|  | *Similarity* | 0.043 | 0.052 | 0.832 | 0.408 |
|  | *Facial Activation * Similarity* | -0.055 | 0.136 | -0.403 | 0.688 |

Table S5. Study 2: Assessing the role of target demographics on pain outcomes

|  | **Variable** | **β** | **SE** | **z/t** | ***p*** |
| --- | --- | --- | --- | --- | --- |
| *Logistic*  *MLM* | *Intercept* | 0.268 | 0.176 | 1.527 | 0.127 |
|  | *Facial Activation* | 9.273 | 0.696 | 13.325 | < .001 |
|  | *Target Race* | -0.188 | 0.174 | -1.080 | 0.280 |
|  | *Target Gender* | -0.509 | 0.348 | -1.463 | 0.144 |
|  | *Facial Activation *Target Race* | -0.135 | 0.651 | -0.207 | 0.836 |
|  | *Facial Activation *Target Gender* | -2.291 | 1.299 | -1.763 | 0.078 |
|  | *Facial Activation *TargetRace*Target Gender* | 0.021 | 0.351 | 0.059 | 0.953 |
| *Linear*  *MLM* | *Intercept* | 4.451 | 0.085 | 52.214 | < .001 |
|  | *Facial Activation* | 3.652 | 0.224 | 16.278 | < .001 |
|  | *Target Race* | 0.079 | 0.171 | 0.461 | 0.645 |
|  | *Target Gender* | 0.171 | 0.171 | 1.004 | 0.317 |
|  | *Facial Activation *Target Race* | -0.241 | 0.451 | -0.534 | 0.594 |
|  | *Facial Activation *Target Gender* | 0.133 | 0.449 | 0.296 | 0.767 |
|  | *Facial Activation *TargetRace*Target Gender* | -0.286 | 0.343 | -0.835 | 0.405 |

Table S6. Study 2: Assessing the role of group membership on pain outcomes

|  | **Variable** | **β** | **SE** | **z/t** | ***p*** |
| --- | --- | --- | --- | --- | --- |
| *Logistic*  *MLM* | *Intercept* | 0.299 | 0.184 | 1.628 | 0.103 |
|  | *Facial Activation* | 9.471 | 0.751 | 12.613 | < .001 |
|  | *Group Race* | -0.353 | 0.368 | -0.957 | 0.339 |
|  | *Group Gender* | 0.528 | 0.366 | 1.443 | 0.149 |
|  | *Facial Activation *Group Race* | -0.548 | 1.409 | -0.389 | 0.697 |
|  | *Facial Activation *Group Gender* | 2.370 | 1.404 | 1.689 | 0.091 |
|  | *Facial Activation *GroupRace* GroupGender* | -0.381 | 0.760 | -0.502 | 0.616 |
| *Linear*  *MLM* | *Intercept* | 4.449 | 0.086 | 51.787 | < .001 |
|  | *Facial Activation* | 3.668 | 0.221 | 16.615 | < .001 |
|  | *Group Race* | 0.093 | 0.192 | 0.483 | 0.630 |
|  | *Group Gender* | 0.070 | 0.172 | 0.404 | 0.687 |
|  | *Facial Activation *Group Race* | -1.236 | 0.496 | -2.492 | 0.014 |
|  | *Facial Activation *Group Gender* | 0.421 | 0.443 | 0.951 | 0.343 |
|  | *Facial Activation *GroupRace* GroupGender* | 0.139 | 0.384 | 0.361 | 0.718 |

Table S7. Study 2: Assessing the role of similarity on pain outcomes

|  | **Variable** | **β** | **SE** | **z** | ***p*** |
| --- | --- | --- | --- | --- | --- |
| *Logistic*  *MLM* | *Intercept* | 0.276 | 0.179 | 1.546 | 0.122 |
|  | *Facial Activation* | 9.321 | 0.704 | 13.233 | < .001 |
|  | *Similarity* | 0.078 | 0.098 | 0.790 | 0.430 |
|  | *Facial Activation * Similarity* | 0.350 | 0.367 | 0.954 | 0.340 |
| *Linear*  *MLM* | *Intercept* | 4.443 | 0.080 | 55.327 | < .001 |
|  | *Facial Activation* | 3.670 | 0.218 | 16.820 | < .001 |
|  | *Similarity* | 0.244 | 0.056 | 4.326 | < .001 |
|  | *Facial Activation * Similarity* | -0.420 | 0.153 | -2.745 | 0.007 |

Table S8. Study 3: Assessing the role of target demographics on pain outcomes

|  | **Variable** | **β** | **SE** | **z** | ***p*** |
| --- | --- | --- | --- | --- | --- |
| *Logistic*  *MLM* | *Intercept* | 0.422 | 0.112 | 3.778 | < .001 |
|  | *Facial Activation* | 7.881 | 0.346 | 22.785 | < .001 |
|  | *Target Race* | -0.066 | 0.222 | -0.300 | 0.764 |
|  | *Target Gender* | -0.244 | 0.221 | -1.103 | 0.270 |
|  | *Facial Activation *Target Race* | -0.781 | 0.657 | -1.188 | 0.235 |
|  | *Facial Activation *Target Gender* | -0.812 | 0.658 | -1.234 | 0.217 |
|  | *Facial Activation *TargetRace*Target Gender* | -0.227 | 0.444 | -0.512 | 0.609 |
| *Linear*  *MLM* | *Intercept* | 4.613 | 0.064 | 72.338 | < .001 |
|  | *Facial Activation* | 3.863 | 0.163 | 23.676 | < .001 |
|  | *Target Race* | -0.076 | 0.128 | -0.596 | 0.551 |
|  | *Target Gender* | 0.115 | 0.128 | 0.899 | 0.370 |
|  | *Facial Activation *Target Race* | 0.098 | 0.327 | 0.298 | 0.766 |
|  | *Facial Activation *Target Gender* | 0.101 | 0.328 | 0.307 | 0.759 |
|  | *Facial Activation *TargetRace*Target Gender* | 0.240 | 0.257 | 0.934 | 0.351 |

Table S9. Study 3: Assessing the role of group membership on pain outcomes

|  | **Variable** | **β** | **SE** | **z** | ***p*** |
| --- | --- | --- | --- | --- | --- |
| *Logistic*  *MLM* | *Intercept* | 0.424 | 0.112 | 3.796 | < .001 |
|  | *Facial Activation* | 7.864 | 0.342 | 22.994 | < .001 |
|  | *Group Race* | -0.446 | 0.251 | -1.775 | 0.076 |
|  | *Group Gender* | 0.407 | 0.227 | 1.794 | 0.073 |
|  | *Facial Activation *Group Race* | -1.587 | 0.742 | -2.139 | 0.032 |
|  | *Facial Activation *Group Gender* | -1.895 | 0.672 | -2.820 | 0.005 |
|  | *Facial Activation *GroupRace* GroupGender* | -0.037 | 0.504 | -0.074 | 0.941 |
| *Linear*  *MLM* | *Intercept* | 4.605 | 0.063 | 72.961 | < .001 |
|  | *Facial Activation* | 3.868 | 0.162 | 23.863 | < .001 |
|  | *Group Race* | -0.056 | 0.142 | -0.390 | 0.697 |
|  | *Group Gender* | 0.414 | 0.130 | 3.177 | 0.002 |
|  | *Facial Activation *Group Race* | -0.463 | 0.365 | -1.268 | 0.206 |
|  | *Facial Activation *Group Gender* | -0.796 | 0.335 | -2.374 | 0.018 |
|  | *Facial Activation *GroupRace* GroupGender* | 0.105 | 0.287 | 0.366 | 0.715 |

Table S10. Study 3: Assessing the role of similarity on pain outcomes

|  | **Variable** | **β** | **SE** | **z** | ***p*** |
| --- | --- | --- | --- | --- | --- |
| *Logistic*  *MLM* | *Intercept* | 0.422 | 0.111 | 3.800 | < .001 |
|  | *Facial Activation* | 7.874 | 0.347 | 22.669 | < .001 |
|  | *Similarity* | 0.167 | 0.076 | 2.179 | 0.029 |
|  | *Facial Activation * Similarity* | 0.024 | 0.230 | 0.104 | 0.917 |
| *Linear*  *MLM* | *Intercept* | 4.606 | 0.061 | 75.095 | < .001 |
|  | *Facial Activation* | 3.877 | 0.161 | 24.114 | < .001 |
|  | *Similarity* | 0.187 | 0.043 | 4.324 | < .001 |
|  | *Facial Activation * Similarity* | -0.288 | 0.113 | -2.548 | 0.011 |

Table S11. Study 4: Assessing the role of target demographics on pain outcomes

|  | **Variable** | **β** | **SE** | **z** | ***p*** |
| --- | --- | --- | --- | --- | --- |
| *Logistic*  *MLM* | *Intercept* | 0.186 | 0.108 | 1.719 | 0.086 |
|  | *Facial Activation* | 7.776 | 0.349 | 22.292 | < .001 |
|  | *Target Race* | -0.278 | 0.213 | -1.303 | 0.193 |
|  | *Target Gender* | -0.097 | 0.214 | -0.451 | 0.652 |
|  | *Facial Activation *Target Race* | 0.170 | 0.650 | 0.261 | 0.794 |
|  | *Facial Activation *Target Gender* | -1.256 | 0.651 | -1.929 | 0.054 |
|  | *Facial Activation *TargetRace*Target Gender* | 0.243 | 0.428 | 0.567 | 0.570 |
| *Linear*  *MLM* | *Intercept* | 5.447 | 0.117 | 46.599 | < .001 |
|  | *Facial Activation* | 0.339 | 0.123 | 2.762 | 0.006 |
|  | *Target Race* | 0.092 | 0.122 | 0.751 | 0.453 |
|  | *Target Gender* | -0.658 | 0.235 | -2.801 | 0.006 |
|  | *Facial Activation *Target Race* | 0.017 | 0.234 | 0.073 | 0.942 |
|  | *Facial Activation *Target Gender* | -0.383 | 0.245 | -1.563 | 0.120 |
|  | *Facial Activation *TargetRace*Target Gender* | 0.583 | 0.469 | 1.242 | 0.216 |

Table S12. Study 4: Assessing the role of group membership on pain outcomes

|  | **Variable** | **β** | **SE** | **z** | ***p*** |
| --- | --- | --- | --- | --- | --- |
| *Logistic*  *MLM* | *Intercept* | 0.175 | 0.107 | 1.635 | 0.102 |
|  | *Facial Activation* | 7.685 | 0.351 | 21.907 | < .001 |
|  | *Group Race* | 0.202 | 0.212 | 0.953 | 0.340 |
|  | *Group Gender* | -0.262 | 0.210 | -1.246 | 0.213 |
|  | *Facial Activation *Group Race* | -0.283 | 0.661 | -0.429 | 0.668 |
|  | *Facial Activation *Group Gender* | -0.587 | 0.656 | -0.895 | 0.371 |
|  | *Facial Activation *GroupRace* GroupGender* | -0.294 | 0.424 | -0.694 | 0.488 |
| *Linear*  *MLM* | *Intercept* | 4.079 | 0.062 | 65.899 | < .001 |
|  | *Facial Activation* | 5.422 | 0.119 | 45.704 | < .001 |
|  | *Group Race* | -0.057 | 0.125 | -0.458 | 0.648 |
|  | *Group Gender* | 0.145 | 0.124 | 1.174 | 0.242 |
|  | *Facial Activation *Group Race* | 0.022 | 0.240 | 0.093 | 0.926 |
|  | *Facial Activation *Group Gender* | -0.135 | 0.237 | -0.569 | 0.570 |
|  | *Facial Activation *GroupRace* GroupGender* | 0.037 | 0.250 | 0.148 | 0.883 |

Table S13. Study 4: Assessing the role of similarity on pain outcomes

|  | **Variable** | **β** | **SE** | **z** | ***p*** |
| --- | --- | --- | --- | --- | --- |
| *Logistic*  *MLM* | *Intercept* | 0.174 | 0.107 | 1.630 | 0.103 |
|  | *Facial Activation* | 7.678 | 0.348 | 22.060 | < .001 |
|  | *Similarity* | -0.104 | 0.075 | -1.392 | 0.164 |
|  | *Facial Activation * Similarity* | -0.414 | 0.229 | -1.812 | 0.070 |
| *Linear*  *MLM* | *Intercept* | 4.079 | 0.062 | 66.107 | < .001 |
|  | *Facial Activation* | 5.421 | 0.118 | 46.071 | < .001 |
|  | *Similarity* | 0.020 | 0.043 | 0.458 | 0.648 |
|  | *Facial Activation * Similarity* | -0.082 | 0.082 | -0.999 | 0.319 |

Table S14. Study 5 rmANOVA of intensity rating means by stimulus emotion category

*Post-hoc for Target Emotion*

| **contrast** |  |  | **estimate** | **SE** | **df** | **t** | **p** |
| --- | --- | --- | --- | --- | --- | --- | --- |
| Neutral | - | Anger | -0.343 | 0.084 | 185 | -4.113 | <.001 |
| Neutral | - | Disgust | -0.720 | 0.106 | 185 | -6.805 | <.001 |
| Neutral | - | Fear | -0.183 | 0.094 | 185 | -1.94 | 0.1078 |
| Neutral | - | Happiness | -1.270 | 0.099 | 185 | -12.843 | <.001 |
| Neutral | - | Pain | -1.859 | 0.111 | 185 | -16.697 | <.001 |
| Neutral | - | Sadness | -0.699 | 0.092 | 185 | -7.623 | <.001 |
| Neutral | - | Surprise | -1.013 | 0.104 | 185 | -9.698 | <.001 |
| Anger | - | Disgust | -0.377 | 0.068 | 185 | -5.53 | <.001 |
| Anger | - | Fear | 0.161 | 0.065 | 185 | 2.482 | 0.0418 |
| Anger | - | Happiness | -0.927 | 0.072 | 185 | -12.862 | <.001 |
| Anger | - | Pain | -1.516 | 0.074 | 185 | -20.505 | <.001 |
| Anger | - | Sadness | -0.355 | 0.064 | 185 | -5.559 | <.001 |
| Anger | - | Surprise | -0.669 | 0.075 | 185 | -8.889 | <.001 |
| Disgust | - | Fear | 0.537 | 0.057 | 185 | 9.375 | <.001 |
| Disgust | - | Happiness | -0.550 | 0.062 | 185 | -8.842 | <.001 |
| Disgust | - | Pain | -1.139 | 0.056 | 185 | -20.299 | <.001 |
| Disgust | - | Sadness | 0.022 | 0.067 | 185 | 0.321 | 0.7488 |
| Disgust | - | Surprise | -0.292 | 0.059 | 185 | -4.928 | <.001 |
| Fear | - | Happiness | -1.087 | 0.068 | 185 | -16.05 | <.001 |
| Fear | - | Pain | -1.676 | 0.072 | 185 | -23.248 | <.001 |
| Fear | - | Sadness | -0.516 | 0.062 | 185 | -8.384 | <.001 |
| Fear | - | Surprise | -0.830 | 0.065 | 185 | -12.708 | <.001 |
| Happiness | - | Pain | -0.589 | 0.058 | 185 | -10.183 | <.001 |
| Happiness | - | Sadness | 0.571 | 0.060 | 185 | 9.596 | <.001 |
| Happiness | - | Surprise | 0.258 | 0.059 | 185 | 4.377 | <.001 |
| Pain | - | Sadness | 1.160 | 0.063 | 185 | 18.506 | <.001 |
| Pain | - | Surprise | 0.847 | 0.062 | 185 | 13.686 | <.001 |
| Sadness | - | Surprise | -0.314 | 0.063 | 185 | -5.012 | <.001 |

*Post-hoc for Target Race*

| **contrast** |  |  |  | **estimate** | **SE** | **df** | **t** | **p** |
| --- | --- | --- | --- | --- | --- | --- | --- | --- |
| Black Targets - White Targets | | | | -0.265 | 0.12 | 185 | -2.212 | 0.0282 |

Table S15. Study 5 rmANOVA of intensity rating means by stimulus facial activation

*Post-hoc for Target Intensity*

| **contrast** |  |  | **estimate** | **SE** | **df** | **t** | **p** |
| --- | --- | --- | --- | --- | --- | --- | --- |
| Facial activation (0%) - Facial activation (20%) | | | 0.089 | 0.076 | 185 | 1.173 | 0.2424 |
| Facial activation (0%) - Facial activation (50%) | | | -0.869 | 0.099 | 185 | -8.746 | < .001 |
| Facial activation (0%) - Facial activation (80%) | | | -1.825 | 0.108 | 185 | -16.942 | < .001 |
| Facial activation (20%) - Facial activation (50%) | | | -0.957 | 0.055 | 185 | -17.438 | < .001 |
| Facial activation (20%) - Facial activation (80%) | | | -1.913 | 0.070 | 185 | -27.431 | < .001 |
| Facial activation (50%) -Facial activation (80%) | | | -0.956 | 0.043 | 185 | -22.352 | < .001 |

Table S16. Study 5 rmANOVA of confidence rating means by stimulus emotion category

*Post-hoc for Target Emotion*

| **contrast** |  |  | **estimate** | **SE** | **df** | **t** | **p** |
| --- | --- | --- | --- | --- | --- | --- | --- |
| Neutral | - | Anger | 0.260 | 0.068 | 185.000 | 3.808 | 0.002 |
| Neutral | - | Disgust | 0.567 | 0.082 | 185.000 | 6.918 | <.001 |
| Neutral | - | Fear | 0.711 | 0.078 | 185.000 | 9.063 | <.001 |
| Neutral | - | Happiness | -0.186 | 0.067 | 185.000 | -2.779 | 0.054 |
| Neutral | - | Pain | -0.082 | 0.074 | 185.000 | -1.100 | 0.546 |
| Neutral | - | Sadness | 0.009 | 0.069 | 185.000 | 0.131 | 0.896 |
| Neutral | - | Surprise | 0.155 | 0.068 | 185.000 | 2.289 | 0.186 |
| Anger | - | Disgust | 0.307 | 0.061 | 185.000 | 5.049 | <.001 |
| Anger | - | Fear | 0.451 | 0.062 | 185.000 | 7.324 | <.001 |
| Anger | - | Happiness | -0.446 | 0.051 | 185.000 | -8.746 | <.001 |
| Anger | - | Pain | -0.342 | 0.060 | 185.000 | -5.696 | <.001 |
| Anger | - | Sadness | -0.251 | 0.061 | 185.000 | -4.133 | 0.001 |
| Anger | - | Surprise | -0.105 | 0.059 | 185.000 | -1.780 | 0.384 |
| Disgust | - | Fear | 0.144 | 0.069 | 185.000 | 2.087 | 0.230 |
| Disgust | - | Happiness | -0.753 | 0.068 | 185.000 | -11.084 | <.001 |
| Disgust | - | Pain | -0.649 | 0.059 | 185.000 | -10.982 | <.001 |
| Disgust | - | Sadness | -0.558 | 0.076 | 185.000 | -7.340 | <.001 |
| Disgust | - | Surprise | -0.412 | 0.073 | 185.000 | -5.651 | <.001 |
| Fear | - | Happiness | -0.897 | 0.067 | 185.000 | -13.468 | <.001 |
| Fear | - | Pain | -0.793 | 0.067 | 185.000 | -11.765 | <.001 |
| Fear | - | Sadness | -0.702 | 0.069 | 185.000 | -10.198 | <.001 |
| Fear | - | Surprise | -0.556 | 0.063 | 185.000 | -8.890 | <.001 |
| Happiness | - | Pain | 0.104 | 0.060 | 185.000 | 1.741 | 0.384 |
| Happiness | - | Sadness | 0.195 | 0.056 | 185.000 | 3.479 | 0.006 |
| Happiness | - | Surprise | 0.341 | 0.055 | 185.000 | 6.222 | <.001 |
| Pain | - | Sadness | 0.091 | 0.059 | 185.000 | 1.549 | 0.384 |
| Pain | - | Surprise | 0.237 | 0.064 | 185.000 | 3.723 | 0.003 |
| Sadness | - | Surprise | 0.146 | 0.064 | 185.000 | 2.269 | 0.186 |

*Post-hoc for Target Gender*

| **contrast** |  |  |  | **estimate** | | **SE** | | **df** | | **t** | | **p** | |
| --- | --- | --- | --- | --- | --- | --- | --- | --- | --- | --- | --- | --- | --- |
| Women Targets - Men Targets | | | -0.218 | | 0.109 | | 185 | | -2 | | 0.0469 | |  |

Table S17. Study 5 rmANOVA confidence rating means by stimulus facial activation

*Post-hoc for Target Intensity*

| **contrast** |  |  | **estimate** | **SE** | **df** | **t** | **p** |
| --- | --- | --- | --- | --- | --- | --- | --- |
| Facial activation (0%) - Facial activation (20%) | | | 0.656 | 0.067 | 185 | 9.761 | < .001 |
| Facial activation (0%) - Facial activation (50%) | | | 0.16 | 0.065 | 185 | 2.469 | 0.0144 |
| Facial activation (0%) - Facial activation (80%) | | | -0.197 | 0.063 | 185 | -3.136 | 0.004 |
| Facial activation (20%) - Facial activation (50%) | | | -0.497 | 0.043 | 185 | -11.579 | < .001 |
| Facial activation (20%) - Facial activation (80%) | | | -0.853 | 0.054 | 185 | -15.852 | < .001 |
| Facial activation (50%) -Facial activation (80%) | | | -0.356 | 0.038 | 185 | -9.427 | < .001 |

*Post-hoc for Target Gender*

| **contrast** |  |  |  | **estimate** | | **SE** | | **df** | | **t** | | **p** | |
| --- | --- | --- | --- | --- | --- | --- | --- | --- | --- | --- | --- | --- | --- |
| Women Targets - Men Targets | | | -0.238 | | 0.11 | | 185 | | -2.155 | | 0.0324 | |  |
