# Supplementary figures and images for: How Pain-Related Facial Expressions Are Evaluated in Relation to Gender, Race, and Emotion
Source: Affect Sci. 2023 Mar 3;4(2):350–69. doi: 10.1007/s42761-023-00181-6 (PMC9982800; doi:10.1007/s42761-023-00181-6)

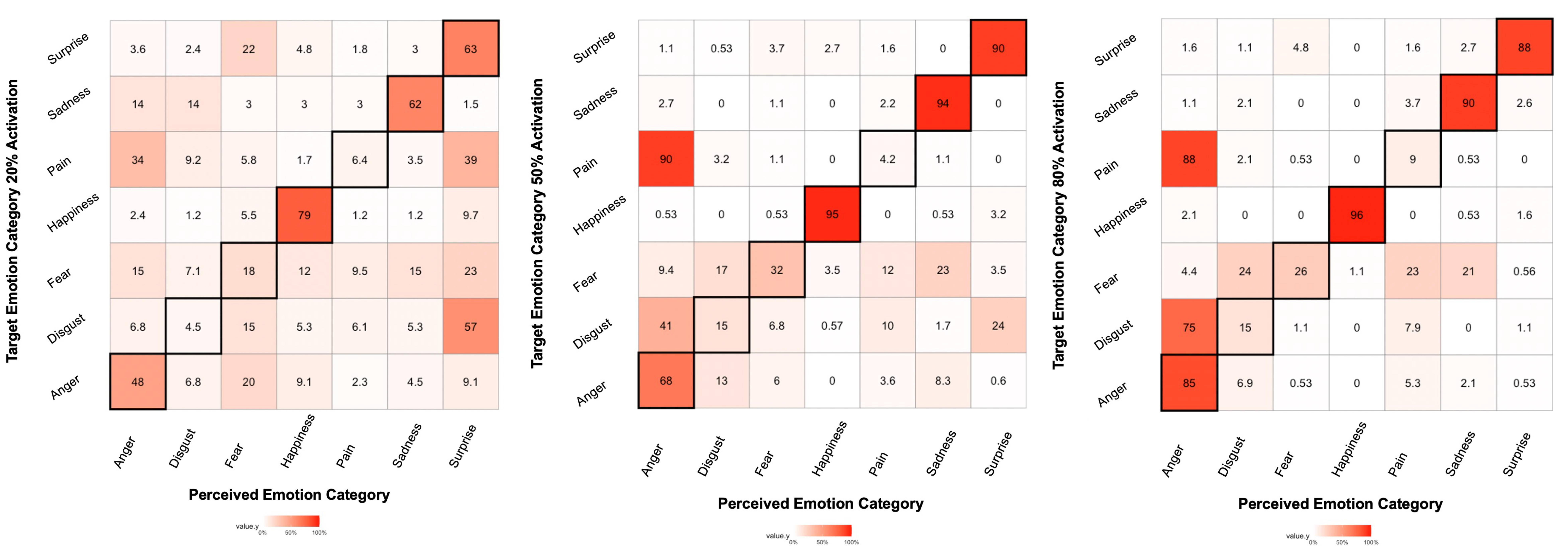

Supplement: Supplementary file 2 — Supplementary file2 (PNG 3775 KB) [file 42761_2023_181_MOESM2_ESM.png]
